# Comammox *Nitrospira* within the Yangtze River continuum: community, biogeography, and ecological drivers

**DOI:** 10.1038/s41396-020-0701-8

**Published:** 2020-06-18

**Authors:** Shufeng Liu, Haiying Wang, Liming Chen, Jiawen Wang, Maosheng Zheng, Sitong Liu, Qian Chen, Jinren Ni

**Affiliations:** 1 Key Laboratory of Water and Sediment Sciences, Ministry of Education, College of Environmental Sciences and Engineering, Peking University, 100871 Beijing, China; 2 Beijing Innovation Center for Engineering Science and Advanced Technology, Peking University, 100871 Beijing, China; 3 Key Laboratory of Regional Energy Systems Optimization, Resources and Environmental Research Academy, North China Electric Power University, 102206 Beijing, China; 4 State Key Laboratory of Plateau Ecology and Agriculture, Qinghai University, 810016 Xining, China

**Keywords:** Environmental sciences, Biogeochemistry, Ecology

## Abstract

The recent discovery of comammox *Nitrospira* as complete nitrifiers has fundamentally renewed perceptions of nitrogen cycling in natural and engineered systems, yet little is known about the environmental controls on these newly recognized bacteria. Based on improved phylogenetic resolution through successful assembly of ten novel genomes (71–96% completeness), we provided the first biogeographic patterns for planktonic and benthic comammox *Nitrospira* in the Yangtze River over a 6030 km continuum. Our study revealed the widespread distributions and relative abundance of comammox *Nitrospira* in this large freshwater system, constituting 30 and 46% of ammonia-oxidizing prokaryotes (AOPs) and displaying 30.4- and 17.9-fold greater abundances than canonical *Nitrospira* representatives in water and sediments, respectively. Comammox *Nitrospira* contributed more to nitrifier abundances (34–87% of AOPs) in typical oligotrophic environments with a higher pH and lower temperature, particularly in the plateau (clade B), mountain and foothill (clade A) areas of the upper reach. The dominant position of planktonic comammox *Nitrospira* was replaced by canonical *Nitrospira* sublineages I/II and ammonia-oxidizing bacteria from the plateau to downstream plain due to environmental selection, while the dissimilarity of benthic comammox *Nitrospira* was moderately associated with geographic distance. A substantial decrease (83%) in benthic comammox *Nitrospira* abundance occurred immediately downstream of the Three Gorges Dam, consistent with a similarly considerable decrease in overall sediment bacterial taxa. Together, this study highlights the previously unrecognized dominance of comammox *Nitrospira* in major river systems and underlines the importance of revisiting the distributions of and controls on nitrification processes within global freshwater environments.

## Introduction

Nitrification is a pivotal process in biogeochemical nitrogen cycling on Earth, transforming 330 and 2000 teragrams of nitrogen per year in global terrestrial and marine environments, respectively [1]. Over the past century, nitrification has generally been perceived as a two-step process catalyzed by separate chemolithoautotrophic nitrifiers: ammonia-oxidizing archaea (AOA) [2] or bacteria (AOB) and nitrite-oxidizing bacteria (NOB) [3]. However, the recent discovery of single microorganisms in the NOB genus *Nitrospira* performing complete oxidation of ammonia to nitrate (termed comammox) [4, 5] has greatly challenged the long-held perspective of two-step nitrification. To date, all the reported comammox bacteria phylogenetically belong to *Nitrospira* sublineage II, the most widely distributed NOB group [6]. The detected comammox *Nitrospira* have been classified into clades A and B based on phylogenies of their ammonia monooxygenases (*amo*) [4, 5], and the clades differ in their ammonia uptake systems [7]. The *amo* operons of comammox *Nitrospira* are phylogenetically distinct from homologs of other nitrifier groups, which could be reliable biomarkers for identification and quantification [4, 5].

As the third group of aerobic ammonia-oxidizing prokaryotes (AOPs), comammox *Nitrospira* have received great attention recently due to their contributions to nitrification. For instance, comammox *Nitrospira* metagenome-assembled genomes (MAGs) have been obtained from multiple engineering environments, including an oil-exploration well [4], a recirculating aquaculture system biofilter [5], a groundwater-fed rapid sand filter [7], several drinking water systems [8, 9], and biological wastewater treatment systems [4, 10], which is of significance for understanding metabolic versatility, ecophysiology, and evolutionary history [7, 11]. Moreover, metacommunity analyses have demonstrated the existence of comammox *Nitrospira* in various natural ecosystems, such as freshwater (clades A and B) [12–14], tidal zone sediments (clade A) [13, 15], and soils (clades A and B) [12–14, 16], with important contributions to ammonia and nitrite oxidation at regional to global scales.

Rivers, as typically lotic ecosystems, are the major channels transferring millions of tons of organic materials and nutrients from the continent to the ocean, contributing substantially to global biogeochemical cycling and energy flow [17, 18]. Large rivers are the cradles of flourishing human civilizations [19]. On the other hand, they are also considered critical sinks for excessive bioavailable nitrogen [20], in which nitrification is coupled with denitrification and anaerobic ammonia oxidation (anammox) to maintain the balance of nitrogen load [21]. Recent studies have confirmed the presence of comammox *Nitrospira* in sediments at specific sites of rivers. For instance, van Kessel et al. [5] obtained comammox-like reads from a few metagenomes in the Tongue River. Black and Just [22] reported an influence of freshwater mussels on comammox *Nitrospira* abundance and function in a backwater region of the upper Mississippi River. Yu et al. [15] illustrated the existence and diversity of complete nitrifiers in estuaries of the Yangtze River. However, investigations of the comammox *Nitrospira* community and the relative contributions of the members to nitrifier abundances are very scarce in large rivers subject to complex natural and anthropogenic influences.

To fill this gap, we implemented synchronous monitoring over a 6030 km continuum along the Yangtze River and provided the first biogeographic patterns of planktonic and benthic comammox *Nitrospira* in the largest river in Asia. Our study not only confirmed the widespread existence of comammox *Nitrospira* but also revealed their significant contributions to the abundances of AOPs and *Nitrospira* genus, driven by environmental preference, particularly in the plateau, mountain, foothill and estuarine areas along the Yangtze River.

## Materials and methods

### Sampling, DNA extraction, and metagenomic shotgun sequencing

**Fig. 1:**
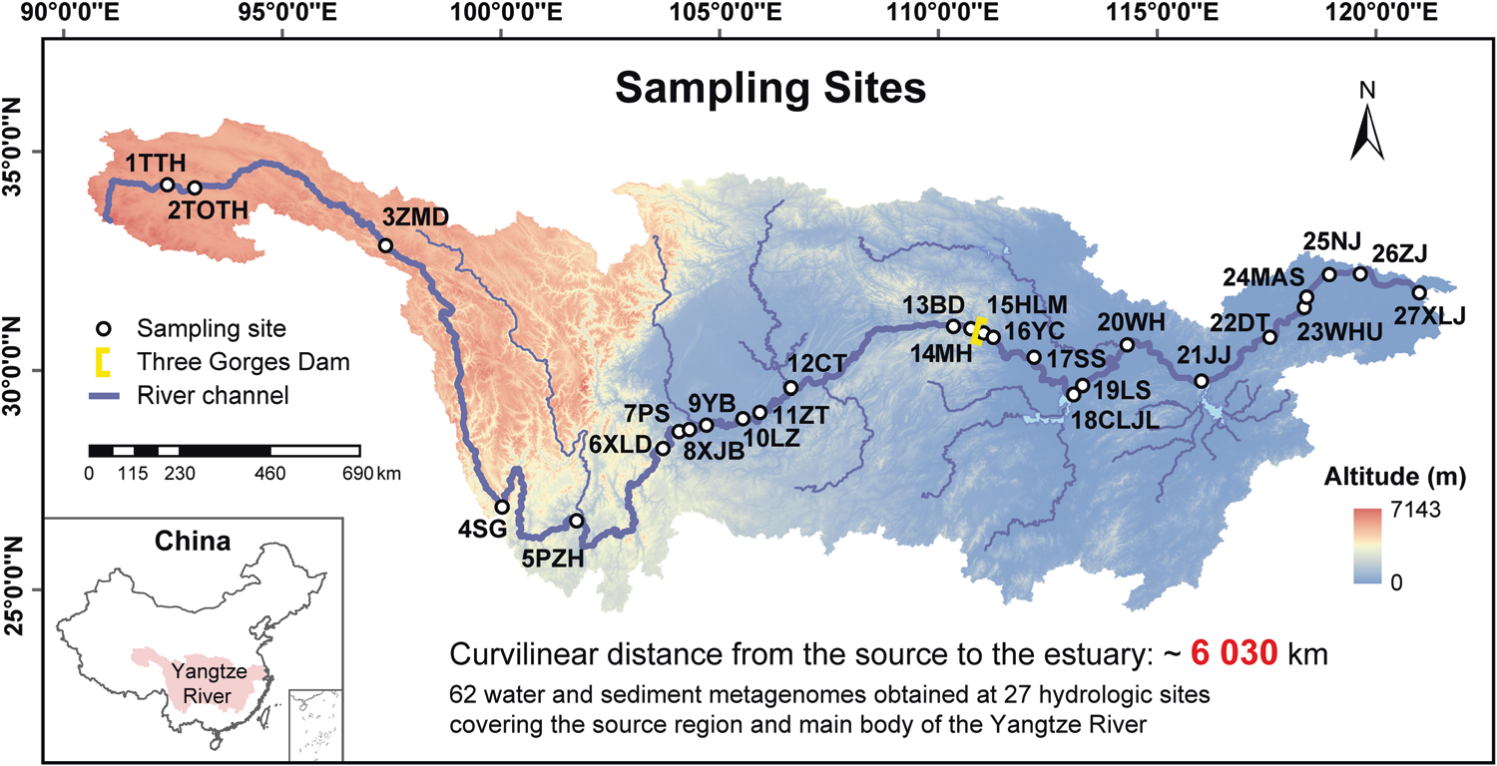
The 27 sampling sites along a 6030 km continuum in the Yangtze River. Sampling sites are numbered sequentially from the source to the estuary. Detailed information about the sampling sites is listed in Supplementary Table S1.

The Yangtze River originates from the Tangula Mountains and flows into the East China Sea, supporting 588 million people in China [23]. In October 2014, paired surface water and sediments were sampled and pretreated synchronously at 24 national hydrologic sites along the mainstream, from Shigu (SG) in the upper reach to Xuliujing (XLJ) in the estuary (Fig. 1; Supplementary Table S1). In July 2017, additional synchronous sampling was implemented at three national hydrologic sites Tuotuohe (TTH), Tongtianhe (TOTH) and Zhimenda (ZMD) in the source region of the Yangtze River located on the Qinghai-Tibetan Plateau. Considering insignificant interannual variations in water quality, riverine habitats and aquatic organisms on the Qinghai-Tibetan Plateau in recent years [24, 25], the above remedial sampling was helpful for obtaining a complete biogeographic pattern of comammox *Nitrospira* throughout the whole river. Details of the sampling were provided in our previous work [25–27]. Spatial and environmental parameters at each site were measured and are summarized in the Supplementary Information. Genomic DNA was extracted from each sample, and then a paired-end strategy (2 × 150 bp with an insert size of 300 bp) was employed for shotgun sequencing on an Illumina HiSeq 4000 platform (Majorbio Company, Shanghai, China) (Supplementary Information). Finally, a total of 30 water and 32 sediment metagenomic datasets were acquired.

### Characterization of the prokaryotic community

All the raw reads were preprocessed using Sickle (v 1.200) (−q 20 −l 50) (https://github.com/najoshi/sickle) and NGS QC Toolkit (v 2.3.3) (−l 70 −s 20) [28]. The number of clean reads in these metagenomic datasets varied from 59.4 to 127.4 million (Supplementary Table S2). Clean reads were annotated by comparisons with the SILVA small subunit database (release 132) using BLASTN (v 2.2.31+) (-max_target_seqs 1 -evalue 10^−20^) [29, 30]. The 16S rRNA gene-like sequences from the BLAST results were assigned to SILVA taxonomies using QIIME (v 1.8.0) [31].

### Construction of an *amoA* amino acid sequence database

A set of *amoA* amino acid sequences of comammox clades A and B, AOA and AOB were collected from the NCBI protein database by searching the key words “ammonia monooxygenase subunit A” and “*amoA*” [4, 5] and then curated manually (Supplementary Information). For public use, the *amoA* sequences and their phylogenetic tree have been deposited in the Figshare database (10.6084/m9.figshare.11346581).

### Read assembly and retrieval of comammox *Nitrospira* MAGs

Clean reads of each metagenomic dataset were searched against the self-constructed database to identify the number of *amoA*-like sequences using a hybrid annotation pipeline (UBLAST and BLASTX) [32] (Supplementary Information). Furthermore, clean reads were de novo assembled into scaffolds individually using IDBA-UD (v 1.1.1) with default parameters [33], and scaffolds longer than 1000 bp were preliminarily assigned to specific taxonomies [34] (Supplementary Information). Then, single-sample binning strategies were conducted on five water and ten sediment assemblies where comammox *Nitrospira* were abundant (i.e., number of comammox *amoA*-like reads >150 and more than 3000 predicted genes assigned to comammox *Nitrospira*). Scaffolds attributed to comammox *Nitrospira* spp. were extracted (Supplementary Information). To separate these composite sequences into individual MAGs, Bowtie2 (v 2.3.0) was applied to calculate coverage values of extracted scaffolds across all the candidate datasets [35], and then MetaBAT2 (v 2.11.2) was utilized to perform single-sample binning by taking tetranucleotide frequency and coverage values into consideration [36]. The completeness and contamination of MAGs were estimated using CheckM (v 1.0.7) [37]. Manual inspections were adopted to reduce contamination according to GC content [8], duplicate presence of 107 essential single-copy genes [38] and redundant single-copy genes identified by CheckM [37] (Supplementary Information). The order and orientation of scaffolds in comammox *Nitrospira* MAGs were corrected by alignments against closely related genomes using the MeDuSa server (http://combo.dbe.unifi.it/medusa) [7, 39]. GapFiller (v 1.10) was used to close a proportion of gaps between scaffolds [40].

### Genome processing

Previously published comammox and canonical *Nitrospira* genomes were downloaded from the NCBI database (https://www.ncbi.nlm.nih.gov/) and mmgenome server (http://madsalbertsen.github.io/mmgenome/). Genome quality was assessed using CheckM [37]. A total of 26 *Nitrospira* genomes (completeness ≥70% with a redundancy ≤10%) downloaded from the available databases (Supplementary Tables S3 and S4), together with the comammox *Nitrospira* MAGs recovered from the Yangtze River, were retained for downstream processing. Genome comparisons were executed using BLAST Ring Image Generator [41]. Gene prediction for all the *Nitrospira* genomes was conducted using Prodigal (v 2.6.3) with the “-p meta” option [42]. The key ammonia oxidation-related genes of each comammox *Nitrospira* were identified according to functional annotation results (Supplementary Information). Gene synteny analysis was performed as described by Palomo et al. [7]. Phylogenetic trees for *amo* operons were constructed using maximum-likelihood estimation. A benchmarked set of 37 elite marker genes (EMGs) with strongly congruent evolutionary histories [43] were extracted, concatenated, aligned, and trimmed for construction of neighbor-joining and maximum-likelihood trees. Detailed procedures of the phylogenetic analyses are summarized in the Supplementary Information. Comammox *Nitrospira* sublineages were defined according to the phylogenetic relationships of their 37 concatenated EMGs. Average nucleotide identity (ANI) values between pairwise *Nitrospira* genomes or 37 concatenated EMGs were computed by OrthoANI (v 0.93.1) [44].

### Evaluation of the comammox *Nitrospira* and canonical nitrifying communities

For comparisons among comammox *Nitrospira*, AOA and AOB, the above self-constructed *amoA* database was supplemented with *amoA* sequences identified in the comammox *Nitrospira* MAGs recovered in this study, forming the final database comprising 45,209 sequences. A hybrid annotation pipeline [32] was utilized to screen clean reads against the final *amoA* database. The hits were normalized by sequencing depth [8, 14] to compare the relative abundances of these AOPs among different samples, which were expressed as the hit number per 10^8^ clean reads. We defined each *amoA* sequence as an AOP taxon. For estimations of comammox *Nitrospira* sublineages and canonical *Nitrospira*, we employed their 37 EMGs (average total length: 17,499 bp) as the reference other than short marker genes (i.e., 16S rRNA and *nxrAB*) for a higher resolution. BBMap (v 37.48) (https://sourceforge.net/projects/bbmap) was applied to perform clean-read mapping for each metagenomic dataset against the 37 EMGs with the top hit saved and minid = 0.9. Sublineage-level relative abundances were inferred from the hit numbers of clean reads normalized by dataset size and the total length of 37 EMGs. To obtain the relative abundances of comammox *Nitrospira* in overall microbial communities, BBMap was employed to search clean reads against all the *Nitrospira* genomes with the top hit saved and minid = 0.9. The relative abundances were calculated from the numbers of clean reads matching comammox *Nitrospira* genomes divided by dataset size [7].

### Statistical analysis

All statistical analyses were executed based on the above relative abundances, using the vegan [45], simba [46], Hmisc [47], and ggplot2 [48] packages and custom scripts in R (v 3.5.0; https://www.r-project.org/). *p* < 0.05 (with 9999 permutations) was considered significant for all statistical tests.

Patterns of community Bray–Curtis dissimilarity of comammox *Nitrospira* and canonical AOPs among different sample groups were visualized using nonmetric multidimensional scaling (NMDS) (“metaMDS” function in vegan). Analysis of similarity (ANOSIM) and one-way analysis of variance (ANOVA) were conducted to test the significance of differences in community structure and relative abundance among specific groups by using the “anosim” and “aov” functions in vegan, respectively. To explore spatial variation in comammox *Nitrospira* versus canonical nitrifiers across landform groups, Mann–Whitney tests (“wilcox.test” function in vegan) were carried out for statistical comparisons.

Matrices of pairwise Bray–Curtis similarities of comammox *Nitrospira* and AOP communities among sites were generated for distance–decay analysis. Mantel tests (“mantel” function in vegan) were used to reveal the significance of Spearman’s rank correlations between community similarity matrices and the geographic distance matrix. Rates of distance decay were calculated as slopes of ordinary least-square regression relationships between ln-transformed geographic distance and ln (*X* + 1)-transformed community similarity [49]. Comparisons of distance–decay slopes among various subsets were performed with a randomization test (“diffslope” function in simba).

Environmental factors usually covary with changes in geographic distance to some extent [49]. To disentangle quantitative effects of geographic and environmental factors on comammox *Nitrospira* and AOP communities, distance-based redundancy analysis (dbRDA) (“capscale” function in vegan) using Bray–Curtis dissimilarity was executed to perform variation partitioning based on a multivariate linear model. The redundancy of environmental factors was assessed to limit strong colinearity (Spearman’s *r*^2^ > 0.7) [49] between variables by using the “varclus” function in Hmisc. Consequently, the nitrate in water was excluded from the subsequent analysis. The variance inflation factors (VIFs) (“vif.cca” function in vegan) of all the remaining environmental factors were less than five, indicating no multicollinearity among them. The “envfit” function in vegan was run to select the significant geographic and environmental factors in the dbRDA. Nonsignificant factors were removed from the first run, and the final results of constrained proportions were reported from the second run by including all the significant factors in the models [49, 50]. The detailed procedures used for variation partitioning have been described elsewhere [50]. Additionally, Spearman’s or Pearson’s correlations were utilized to identify niche preferences for significant environmental factors between comammox *Nitrospira* and canonical nitrifiers and comammox clades A and B. Mann–Whitney tests were conducted to explore the spatial dependency of significant environmental variables among distinct landform groups.

To investigate the impacts of large dams, principal coordinate analysis (PCoA) (“prcomp” function in vegan) and ANOSIM based on Bray–Curtis dissimilarity were applied to test for a significant difference in the AOP communities immediately upstream and downstream of the Three Gorges Dam (TGD). One-way ANOVA was utilized to test for a significance difference in the relative abundances of comammox *Nitrospira* and canonical AOPs.

## Results

### Overall prokaryotic compositions

We obtained the overall taxonomic profiles of prokaryotes in the Yangtze River. Within all the 16S rRNA gene-like sequences, *Bacteria* were the most abundant domain (water, 99.7 ± 0.2%; sediments, 97.6 ± 1.6%). At the phylum level (Supplementary Fig. S1a and b), *Proteobacteria*, *Bacteroidetes*, *Firmicutes*, *Actinobacteria*, *Elusimicrobia*, and *Acidobacteria* dominated the prokaryotic communities, with overall relative abundances of 90.6 ± 3.9% and 74.3 ± 6.0% in water and sediments, respectively. Here, the phylum *Nitrospirae* accounted for 0.5 ± 0.4% of the planktonic prokaryotes and 1.9 ± 0.9% of the benthic prokaryotes. At the genus level (Supplementary Fig. S1c, d), *Streptococcus*, *Endomicrobium*, *Klebsiella,* and *Myroides* were the most abundant groups, constituting 19.5 ± 0.7% and 18.8 ± 0.8% of planktonic and benthic prokaryotes, respectively.

### Genome reconstruction of novel comammox *Nitrospira*

To reconstruct comammox *Nitrospira* MAGs, we conducted single-sample assembly and binning strategies on metagenomic datasets along the Yangtze River. Ten novel comammox *Nitrospira* MAGs (71–96% completeness with a low contamination rate of ≤5%) were reconstructed with metagenomes from fluvial water in the upper reach and surface sediments from the middle reach to the estuary (Table 1). The genome sizes of the recovered MAGs ranged from 2.6–3.7 megabases (Mb). BLAST comparisons showed that the nitrification-related genes shared high similarities with those of *Nitrospira inopinata*, the only complete nitrifier isolated to date [51] (Supplementary Fig. S2). Phylogenetic analysis of available *amo* operons revealed that all the retrieved comammox *Nitrospira* belonged to clade A (Supplementary Fig. S3). For eight MAGs from the Yangtze River, the *amo* and either hydroxylamine dehydrogenase or cytochrome *c* maturation genes of the ammonia-oxidation pathway were located in the proximate genomic region (Supplementary Fig. S4). These results were similar to those reported previously except for *N. inopinata* [7]. In addition, new gene arrangements were reported in the Yangtze comammox *Nitrospira*. In close proximity to the regions of ammonia-oxidation gene clusters, seven MAGs encoded one to three peptidases for deamination, three (named YR-XLD, YR-PS, and YR-XJB) of which encoded an additional cupredoxin for copper homeostasis in ammonia oxidation, and the other three (YR-WHU, YR-XLJ-2, and YR-XLJ-3) of which harbored an extra gene cluster of *fkpA-*cytochrome *c-nirK*, putatively functioning in periplasmic nitrite reduction to nitric oxide (NO). Adjacent gene organizations might confer corresponding comammox *Nitrospira* with concurrent evolution, increasing horizontal transfer and stable coexpression of these functions [52].

**Table 1:** Genomic features of the ten novel comammox *Nitrospira* MAGs reconstructed with metagenome assemblies in the Yangtze River.

Features	YR-XLD^a^	YR-PS^a^	YR-XJB^a^	YR-LS^a^	YR-JJ^a^	YR-WHU^a^	YR-ZJ^a^	YR-XLJ-1^a^	YR-XLJ-2^a^	YR-XLJ-3^a^
Size (Mb)	3.58	3.28	3.69	3.58	2.68	3.39	3.33	3.55	2.95	2.77
No. of scaffolds	238	226	224	465	251	363	572	145	116	118
N50 value (bp)	3,5950	3,3452	3,8369	1,7570	2,3791	2,9392	1,1301	27,8793	4,4739	3,8459
GC content (%)	56.1	55.9	55.9	57.8	56.1	55.7	56.0	55.7	58.3	57.2
No. of coding genes	4039	3733	4093	4082	3072	4033	3898	3859	3085	2877
No. of tRNAs^b^	37	35	37	44	35	43	39	40	44	39
No. of genes annotated by KO^c^	1737 (43.0%)	1588 (42.5%)	1686 (41.2%)	1873 (45.9%)	1435 (46.7%)	1808 (44.8%)	1746 (44.8%)	1617 (41.9%)	1357 (41.3%)	1339 (46.5%)
No. of genes annotated by COG^d^	2831 (70.1%)	2602 (69.7%)	2845 (69.5%)	2994 (73.3%)	2257 (73.5%)	2904 (72.0%)	2778 (71.3%)	2681 (69.5%)	2228 (72.2%)	2113 (73.4%)
Completeness (%)	74.0	71.0	76.2	90.0	74.5	76.1	83.8	91.0	88.2	95.2
Contamination (%)	4.6	1.7	5.0	4.6	0.9	2.5	2.1	2.7	4.1	3.7
Comammox *N*. sublineages^e^	A-Ic	A-Ic	A-Ic	A-Ib	A-Ic	A-Ic	A-IIb	A-IIa	A-Ib	A-Ic
*amoA*	+	+	+	+	+	+	−	+	+	+
*amoB*	+	+	+	+	+	+	+^f^	+	+	+
*amoC*	+	+	+^f^	+	+	+	+^f^	+^f^	+	+
*hao*	+	−	+	+	+	+	+	+	+	−
*nxrA*	+	+	+	+	−	+	+	+	+	+
*nxrB*	+	+	+	+	+	+	+	+	+	+

^a^The comammox *Nitrospira* MAGs YR-XLD, YR-PS and YR-XJB were reconstructed with metagenome assemblies from the water, while other MAGs were reconstructed with metagenome assemblies from the sediments.

^b^The tRNAs were predicted by the tRNAscan-SE server (http://lowelab.ucsc.edu/tRNAscan-SE/).

^c^KO: KEGG Orthology.

^d^COG: Clusters of Orthologous Groups.

^e^The comammox *Nitrospira* sublineages were reclassified in Supplementary Fig. S5.

^f^Identified *amoB* or *amoC* operons were not in the *amo* clusters, but at some other loci in the MAG.

+: Gene was recovered from the corresponding MAG.

−: Gene was not recovered from the corresponding MAG possibly due to scaffold fragmentations.

To investigate the phylogenetic positions of recovered MAGs in *Nitrospira*, 37 concatenated EMGs were used to construct neighbor-joining and maximum-likelihood trees (Supplementary Fig. S5). It was found that all the comammox *Nitrospira* fell into *Nitrospira* sublineage II. Within the phylogenetic trees, ten comammox *Nitrospira* MAGs from the Yangtze River were located on four different branches. Seven Yangtze MAGs, together with *N. inopinata*, SG-bin2 and ST-bin4, formed a basal sublineage that was divergent from the other one (containing ten MAGs) within comammox clade A, with robust bootstrap support (≥60) on branch nodes. To update their taxonomic affiliations, we separated comammox clade A into two sublineages, namely, clades A-I and A-II. These two clades could be further divided into A-Ia, A-Ib, A-Ic, A-IIa and A-IIb, respectively, with convincing support (>75) for divergence. Based on the genome-wide ANI, the clade-A genomes constituted 14 separate species (at a species-level cutoff of ~95% ANI [53]), including four novel species from the Yangtze River (three in clade A-I and one in clade A-II) (Supplementary Fig. S6). Moreover, the maximum intersublineage ANI between the A-I and A-II genomes was 74.6%. A-Ic shared a maximum intersublineage ANI of 86.9% and 74.0% with A-Ib and A-Ia, respectively, and A-IIa shared a maximum intersublineage ANI of 76.6% with A-IIb. These findings indicated that the reclassified sublineages in comammox clade A were sufficiently divergent from one another to be meaningful. In view of the limited existing comammox *Nitrospira* genomes (Supplementary Tables S3 and S4), the phylogenetic resolution was improved through the novel genomes extracted in the present study.

### Biogeographic patterns of comammox *Nitrospira*

Based on the established phylogenies, we further explored the biogeographic patterns of comammox *Nitrospira* along the Yangtze River. All the comammox *Nitrospira* genomes recruited an average of 0.25% (0.02–1.4%) and 0.36% (0.004–2.9%) clean reads in water and sediments, respectively (Supplementary Fig. S7). As critical marker genes, *amoA* and 37 EMGs recruited a sum of 9 452 and 75 357 clean reads for comammox *Nitrospira*, respectively, and the numbers of clean reads obtained using the two methods were strongly correlated (Supplementary Fig. S8). Comammox-affiliated reads were detected in water and sediment metagenomes at all sites, suggesting the wide occurrence of comammox *Nitrospira* along the whole Yangtze River.

**Fig. 2:**
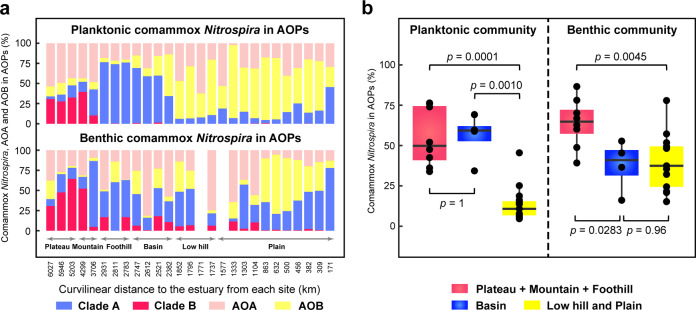
Biogeographic patterns for comammox *Nitrospira* and canonical AOPs. **a** Spatial variation in the percentage of comammox *Nitrospira* within AOPs from the headwater to the estuary. **b** Effects of landforms on the percentage of comammox *Nitrospira* within AOPs. The *p* values of Mann–Whitney tests are provided. All the relative abundance metrics used are based on the normalized *amoA* gene hits.

Comammox *amoA* sequences outnumbered those of either AOA or AOB in water from 9 sites (out of 27) and sediments from 14 sites (out of 25), and planktonic and benthic comammox *Nitrospira* separately comprised an average of 30% (3.8–76.4%) and 46% (14.5–86.6%) of AOPs (Fig. 2a). This suggested a significant abundance contribution of comammox *Nitrospira* to the AOPs in the Yangtze River. NMDS was applied to depict habitat-specific patterns across all the metagenomes (Supplementary Fig. S9). Clear separation of the comammox community was observed not only between samples in the source area and main body of the Yangtze River (ANOSIM: *r* = 0.63, *p* = 0.0001) but also between water and sediment groups. The compositional differentiation of comammox *Nitrospira* (ANOSIM: *r* = 0.32, *p* = 0.0001) between water and sediments of the Yangtze River was much stronger than that of AOA and AOB (ANOSIM: *r* = 0.25 and 0.07, *p* = 0.0001 and 0.0076, respectively). Comammox *Nitrospira*, rather than canonical AOPs, were significantly enriched in riverine sediments (one-way ANOVA, *p* = 0.008) (Supplementary Fig. S10), which might suggest their microaerophilic adaptability and attached-growth preference [7, 11, 54]. Moreover, macroscopic landforms had critical impacts on spatial variation in comammox *Nitrospira* and canonical AOPs (Fig. 2b). For the planktonic and benthic communities, comammox *Nitrospira* accounted for significantly larger proportions (33.9–86.6%) of the AOPs in the plateau/mountain/foothill areas than in the low hill and plain (Mann–Whitney tests, *p* = 0.0001–0.0045). Particularly, clade B dominated the comammox community in the plateau area, constituting 27.7–64.4% of planktonic and benthic AOPs, while clade A outcompeted clade B from the mountain to plain areas. The canonical AOPs were more abundant than comammox *Nitrospira* in most water and sediment samples from the low hill and plain, where *Nitrosoarchaeum*, *Nitrosopumilus*, *Nitrosospira*, *Nitrosovibrio* and *Nitrosomonas* were the major genera (Supplementary Tables S5 and S6). Notably, comammox *Nitrospira* were found to be more abundant than AOA or AOB at estuarine site XLJ (Fig. 2a). This finding was not consistent with those derived from PCR assays in similar estuarial environments [13, 15].

**Fig. 3:**
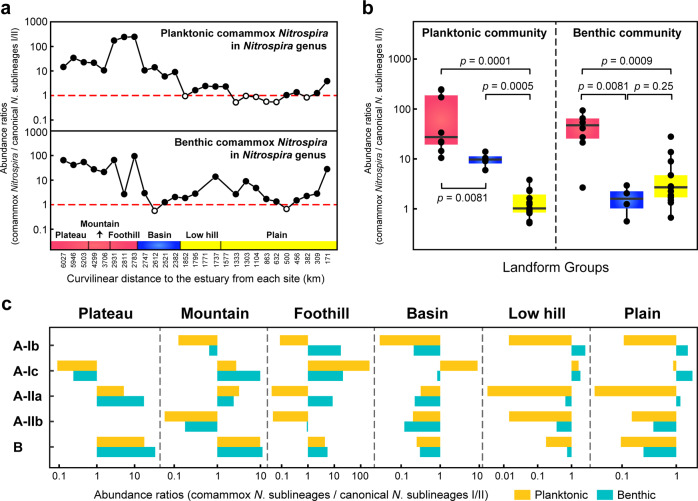
Biogeographic patterns for comammox and canonical *Nitrospira*. **a** Spatial variation in the abundance ratio of comammox and canonical *Nitrospira* representatives from the headwater to the estuary. Black dots indicate that comammox *Nitrospira* outcompete canonical *Nitrospira* sublineages I/II in abundance. **b** Effects of landforms on the abundance ratios of comammox *Nitrospira* and canonical *Nitrospira* sublineages I/II. The *p* values of Mann–Whitney tests are provided. **c** Abundance ratios of five dominant comammox *Nitrospira* sublineages and canonical *Nitrospira* sublineages I/II corresponding to specific landforms. All the relative abundance metrics used are based on the normalized reads mapped to 37 EMGs.

All the metagenomes contained an average of 1216 reads (11–8416) affiliated with the comammox *Nitrospira* EMGs (Supplementary Fig. S8). Comammox *Nitrospira* were more abundant than canonical *Nitrospira* sublineages I/II in water samples from 20 sites (out of 27) (average: 30.4-fold) and sediments from 23 sites (out of 25) (average: 17.9-fold), suggesting that the comammox community contributed greatly to *Nitrospira* abundance and nitrite oxidization in the Yangtze River (Fig. 3a). For the planktonic communities, comammox *Nitrospira* in the plateau/mountain/foothill and basin landforms of the upper reach were in higher relative abundance than in the low hill and plain areas (Mann–Whitney tests, *p* = 0.0001–0.0081), as they mostly outnumbered representatives of canonical *Nitrospira* sublineages I/II 5.9–246.4 fold from the plateau to basin (Fig. 3b). Similarly, benthic comammox *Nitrospira* contributed more to *Nitrospira* abundance in the plateau/mountain/foothill than in the basin, low hill and plain areas (Mann–Whitney tests, *p* = 0.0009–0.0081), i.e., 2.7–93.4-fold more abundant than the representatives of canonical *Nitrospira* sublineages I/II from the plateau to foothill (Fig. 3b). The opposing trends for dominant comammox *Nitrospira* sublineages were also obtained (Fig. 3c). Comammox clade B was much more abundant than clade A in both water (average: 3.3-fold) and sediments (average: 2.7-fold) on the plateau. Clade A-IIa was the most abundant clade-A sublineage on the plateau, and clade-A-Ic species recruited far more reads than other comammox *Nitrospira* sublineages from the foothill to estuarine areas, except for sediments of the low hill area (clade A-Ib). The mountainous environments harbored multiple clades (A-Ic, A-IIa, and B) as the dominant comammox *Nitrospira* sublineages, each of which was much more abundant than canonical *Nitrospira* sublineages I/II. However, comammox clade A-Ia, with *N*. *inopinata* as the only representative, was not detected in the water or sediments of the Yangtze River.

### Effects of geographic distance and environmental factors

**Fig. 4:**
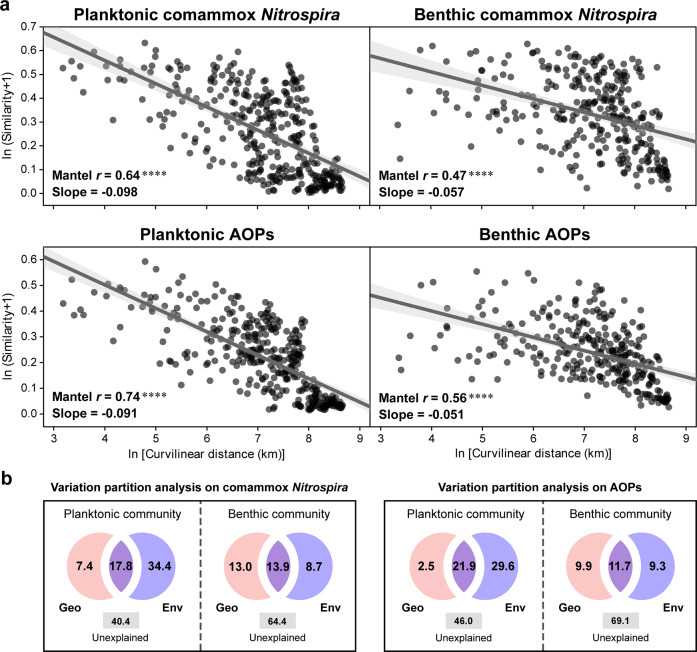
Spatial turnover of comammox *Nitrospira* and AOPs. **a** Distance–decay curves of comammox *Nitrospira* and AOP community Bray–Curtis similarity versus curvilinear distance for all the water and sediment samples from the Yangtze River. The gray lines indicate the ordinary least square linear regressions across all the samples in each habitat. The shaded areas represent 95% confidence intervals. Mantel Spearman’s rank correlation coefficients (*r*) and slopes are provided. Asterisks denote the significance of correlations (****p = 0.0001). More statistics are summarized in Supplementary Table S7. **b** Distance-based redundancy analysis (dbRDA) decoupling the pure and joint effects (as a percentage) of geographic distance (Geo) and environmental factors (Env) on the *β*-diversity of comammox *Nitrospira* and AOP communities. All the relative abundance metrics used are based on the normalized *amoA* gene hits.

First, distance–decay relationships are comprehensive measurements of biogeography for communities from all domains of life [49]. Significant declines in comammox *Nitrospira* and AOP community similarities with increasing geographic (curvilinear) distance were demonstrated for water and sediment samples throughout the Yangtze River (Mantel Spearman’s *r* = 0.47–0.74, *p* = 0.0001) (Fig. 4a; Supplementary Table S7). The slopes that were assessed by linear regression models for planktonic comammox *Nitrospira* (-0.098) and AOPs (-0.091) were significantly steeper than the corresponding values (−0.057 and −0.051, respectively) of the benthic samples (*p* = 0.0001). Steeper distance–decay slopes indicated higher spatial turnover rates of communities [49].

Second, we examined the extent to which geographic distance and environmental factors explained the biogeography of comammox *Nitrospira* and AOPs in the Yangtze River. For the planktonic communities, considerably larger proportions of comammox *Nitrospira* (59.6%) and AOP (54.0%) community variabilities were explained by both geographic distance and environmental factors in the dbRDA (Fig. 4b; Supplementary Table S8). Among these, up to 52.2% and 51.5% of comammox *Nitrospira* and AOP variation, respectively, was attributed to the total effects of environmental variables, whereas geographic distance explained only 25.2% and 24.4%. The pure effects of environmental factors (29.5–34.4%) were much larger than those of geographic distance (2.5~7.4%). These results suggested that environmental filtering, as a proxy of deterministic processes, played a critical role in the distance–decay patterns of the planktonic comammox *Nitrospira* and AOPs. Nevertheless, the dissimilarity of the benthic comammox community was fairly constrained by geographic distance (26.9%) (Fig. 4b; Supplementary Table S9). A range of 40.4–69.1% of variation was not explained by the selected factors, which might be ascribed to unmeasured variables, biotic interactions, and drift processes [55].

**Fig. 5:**
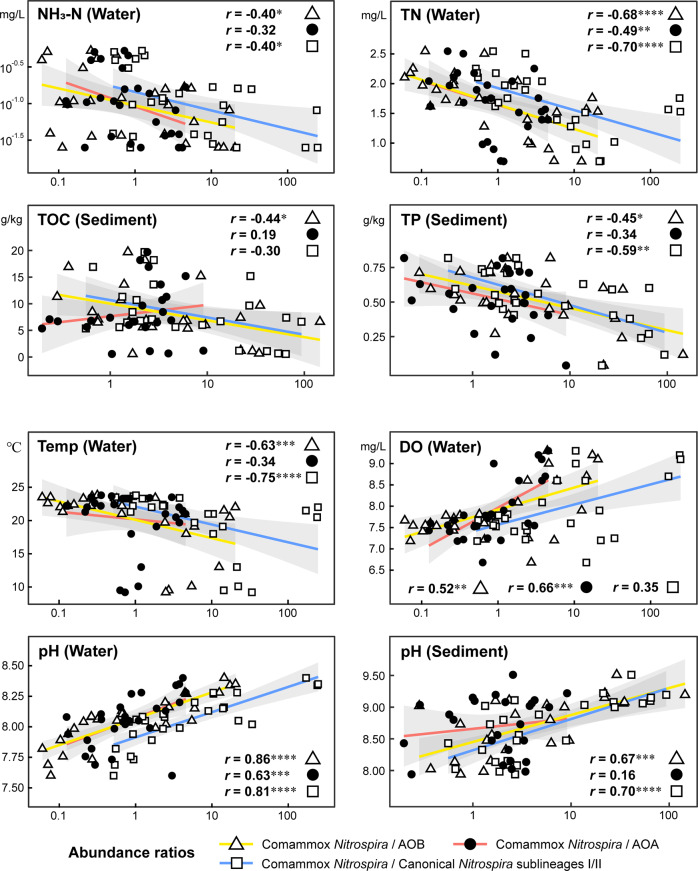
Environmental drivers of niche separation among comammox *Nitrospira* and canonical nitrifiers. Spearman’s correlations (*r*) between the significant environmental factors (selected in the dbRDA) and abundance ratios of comammox *Nitrospira* and canonical nitrifiers are provided. Asterisks denote the significance of correlations (****p = 0.0001, ***0.0001 < *p* < 0.001, **0.001 < *p* < 0.01 and *0.01 < *p* < 0.05). Colored lines indicate the ordinary least square linear regressions, with the shaded area representing 95% confidence intervals. NH_3_-N ammonia, TN total nitrogen, TOC total organic carbon, TP total phosphorus, Temp temperature, DO dissolved oxygen. The abundance ratios of comammox *Nitrospira* and AOB or AOA are based on the normalized *amoA* gene hits. The abundance ratios of comammox *Nitrospira* and canonical *Nitrospira* sublineages I/II are based on the normalized reads mapped to 37 EMGs.

Third, we investigated specific associations between significant environmental factors and spatial variation in comammox *Nitrospira* and canonical nitrifiers. The dbRDA showed that dissimilarities of both comammox *Nitrospira* and AOPs responded significantly to gradients of nutrients such as total nitrogen (TN) (*p* = 0.0001) in water and total organic carbon (TOC) (*p* = 0.0022–0.0053) and total phosphorus (TP) (*p* = 0.0100~0.0440) in sediments (Supplementary Tables S8 and S9). Ammonia (NH_3_–N) concentration in water was found to significantly (*p* = 0.0260) influence planktonic AOP assemblages rather than the comammox community. In detail, the abundance ratio of comammox clades A and B was positively correlated with TN (Pearson’s adjusted *R*^2^ = 0.20, *p* = 0.0121) and TOC (Pearson’s adjusted *R*^2^ = 0.25, *p* = 0.0058) (Supplementary Fig. S11). We also found that the abundance ratios of comammox *Nitrospira* and AOB or canonical *Nitrospira* sublineages I/II were strongly and negatively correlated with NH_3_-N, TN, TOC, or TP (Spearman’s *r* = −0.40 to −0.70, *p* = 0.0001–0.0406), whereas the abundance ratio of comammox *Nitrospira* and AOA was not significantly correlated with NH_3_-N, TOC, and TP (*p* = 0.10–0.35) and less significantly related to TN when comparing Spearman’s correlation coefficients (Fig. 5). Notably, the plateau/mountain/foothill areas possessed significantly lower NH_3_-N, TN, TOC, and TP concentrations than the low hill and plain regions (Mann–Whitney tests, *p* = 0.0001~0.0327) (Supplementary Fig. S12), indicating a greater preference of comammox *Nitrospira* for relatively oligotrophic environments than of canonical *Nitrospira* and AOB in the upper reach of the Yangtze River. Additionally, temperature, pH and dissolved oxygen (DO) were also the key factors shaping biogeography (*p* = 0.0001–0.0125) (Supplementary Tables S8 and S9). The abundance ratios of comammox clades A and B positively correlated with the above three factors in water (Pearson’s adjusted *R*^2^ = 0.27–0.54, *p* = 0.0001–0.0034) (Supplementary Fig. S9). The abundance ratios of planktonic comammox *Nitrospira* and AOB or canonical *Nitrospira* sublineages I/II exhibited significantly negative correlations with temperature (Spearman’s *r* = −0.63 to −0.75, *p* = 0.0001–0.0004), while the abundance ratio of planktonic comammox *Nitrospira* and AOA displayed the most significantly positive correlation with DO (Spearman’s *r* = 0.66, *p* = 0.0002) (Fig. 5). We also found that the abundance ratios of comammox *Nitrospira* and canonical nitrifiers were all significantly and positively correlated with pH in water and sediments (Spearman’s *r* = 0.62–0.86, *p* = 0.0001–0.0006), except for that of benthic comammox *Nitrospira* and AOA (Fig. 5). The plateau/mountain/foothill areas had a higher pH and lower temperature than the low hill and plain areas (Mann–Whitney tests, *p* = 0.0002–0.0016) (Supplementary Fig. S12), indicating that comammox *Nitrospira*, rather than canonical *Nitrospira* and AOB, had large advantages in residing in relatively cold and alkalescent environments of the upper Yangtze River.

### Impacts of the Three Gorges Dam

**Fig. 6:**
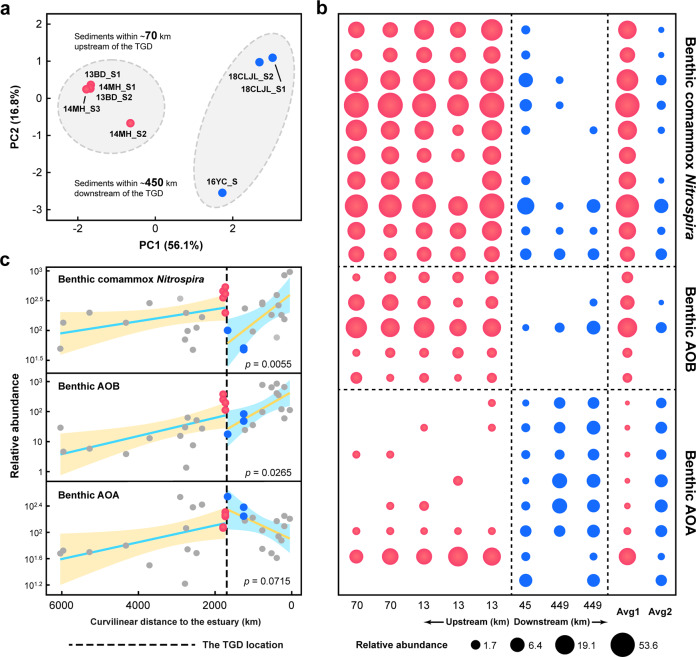
Local influence of the Three Gorges Dam (TGD) on benthic comammox *Nitrospira* and canonical AOPs. **a** Principal coordinate analysis (PCoA) showing compositional differences in AOPs between sediments collected immediately upstream (~70 km) and downstream (~450 km) of the TGD. **b** Bubble diagram revealing significant differences (one-way ANOVA, *p* < 0.05) in dominant taxa (*amoA*-related abundance ≥3 in at least one sample) between sediments collected immediately upstream and downstream of the TGD. Detailed information on each dominant taxon is summarized in Supplementary Table S10. **c** Spatial variation in the relative abundances of comammox *Nitrospira* and canonical AOPs in separate reaches upstream (site TTH to MH) and downstream (site YC to XLJ) of the TGD. Colored lines indicate the ordinary least square linear regressions, with the shaded areas representing 95% confidence intervals. The *p* values of one-way ANOVA are provided for the significance of the difference in abundance between sediments collected immediately upstream and downstream of the TGD. All the relative abundance metrics used are based on the normalized *amoA* gene hits.

Artificial damming splits the river channel into separate reaches and alters the microbial ecology immediately upstream and downstream [25–27]. The TGD, as one of the world’s largest dams, had a profound influence on the spatial succession of comammox *Nitrospira* and canonical AOPs in sediments. To examine the dam effects, only the sites mostly affected by the TGD were chosen. The PCoA revealed that benthic AOP communities at sites Yichang (YC) and Chenglingjilian (CLJL) were apparently distinct from those at sites Badong (BD) and Miaohe (MH) (Fig. 6a). The consistency of these results was further confirmed by ANOSIM (*r* = 0.88, *p* = 0.0180). Moreover, multiple dominant comammox *Nitrospira* taxa (clades A-Ib, A-Ic, A-IIb, and B), constituting an average of 31.1% of the AOPs in upstream sediments of the TGD, exhibited a significant decrease in relative abundance downstream of the TGD (one-way ANOVA, *p* = 0.0007–0.0366) (Fig. 6b; Supplementary Table S10). Similar variation was also observed for abundant AOB taxa (*Nitrosomonas*), whereas some AOA taxa belonging to *Crenarchaeote*, *Nitrososphaera* and *Nitrosarchaeum* had a higher relative abundance immediately downstream of the TGD (Fig. 6b; Supplementary Table S10). From immediately upstream to downstream of the TGD, the relative abundance of benthic comammox *Nitrospira* and AOB decreased by 83.0% and 78.0%, respectively (one-way ANOVA, *p* = 0.0055–0.0265) (Fig. 6c). As a result, AOA were suspected to play a dominant role in the first step of nitrification in sediments immediately downstream of the TGD (average 69.5% of AOPs).

## Discussion

Recent advances unraveling new biological features of comammox have upended our perspectives on nitrogen-cycling microbes, and the relative contributions of comammox *Nitrospira* to nitrifier abundances must be evaluated in various habitats [56]. Here, we presented the first biogeography of planktonic and benthic comammox *Nitrospira* along a 6030 km continuum of the Yangtze River, which was interpreted in terms of natural and anthropogenic influences.

Most comammox *Nitrospira* MAGs have been extracted from bacterial assemblies in engineered ecosystems where *Nitrospira* are enriched [4, 5, 7–10]. We recovered ten substantially or nearly complete comammox clade-A MAGs from riverine water and sediments that harbored complex bacterial assemblages [26], providing new insights into comammox community habitats. The genetic operons for ammonia- and nitrite-oxidation pathways were detected in all the recovered MAGs, and the newly reported gene arrangements might confer comammox *Nitrospira* specific functions. The peptidases in proximity to the ammonia-oxidation gene clusters might confer YR-XLD, YR-PS, and YR-XJB a competitive advantage in peptide decomposition for ammonia oxidation over other comammox *Nitrospira*. Similarly, the *fkpA*-cytochrome *c*-*nirK* in proximity to the ammonia-oxidation genes might confer YR-WHU, YR-XLJ-2, and YR-XLJ-3 an advantage in producing NO efficiently accompanied by ammonia oxidation. Kinetic studies have demonstrated that *N*. *inopinata* produces NO during ammonia oxidation under oxic conditions, and lower NH_3_–N concentrations lead to significantly less NO generation [57]. Moreover, based on the concatenated EMGs allowing evolutionary history to be elucidated with confidence [43], the phylogenetic and taxonomic affiliations of comammox clade A were improved by dividing them into small-scale sublineages, though representative species in the sublineages (A-Ia and A-Ib) were limited in the present study. The 16S rRNA-, *nxrA*-, and *nxrB*-based methods could not distinguish comammox *Nitrospira* from canonical *Nitrospira*, which was one of the reasons for the long-term overlook of comammox [4, 5, 9, 56]. Genome reconstructions of novel MAGs belonging to clades A-Ib, A-Ic, A-IIa, and A-IIb from the Yangtze River expanded comammox *Nitrospira* diversity to facilitate further research on biogeography in large rivers.

The coexistence of comammox *Nitrospira*, canonical AOPs, and strict NOB for cross-feeding interactions has been illustrated in natural and engineered ecosystems [4, 5, 7–10, 22, 58], raising new hypotheses about the abundance and roles of complete nitrifiers. Apart from small-scale river reaches [5, 22] and estuaries [13, 15], comammox *Nitrospira* appeared ubiquitously in water and sediments along the Yangtze River. Within ammonia-oxidation guilds, planktonic and benthic comammox *Nitrospira* accounted for 30% and 46% of AOPs, respectively, and dominated the AOPs at many sites. A previous study indicated that comammox *Nitrospira* outnumbered AOB in only two riverine sediments [13], and other natural environments, such as paddy soils [13], subtropical forest soils [16], and intertidal sediments [13, 15], were reported to harbor abundant AOA or AOB, having a several-orders-of-magnitude higher abundance than the comammox community. Comammox *Nitrospira* have already been observed to be the most dominant AOPs in engineered environments, including drinking water systems [8], rapid gravity sand filters [58], recirculating aquaculture systems [59] and wastewater treatment plants [13]. Within nitrite oxidizers, comammox *Nitrospira* were observed to be much more abundant than representatives of canonical *Nitrospira* sublineages I/II in most areas of the Yangtze River. Comammox *Nitrospira* probably do not diffuse nitrite outside the cell at low ammonia concentrations [4], which might reduce the available nitrite for strict NOB nearby. Large proportions of comammox guilds within the *Nitrospira* genus have also been estimated for groundwater wells (58–74%) [4], groundwater-fed rapid sand filters (28–100%) [60] and biological nutrient removal systems (42–71%) [4, 10]. Our results indicated that the majority of the *Nitrospira* members detected in riverine ecosystems were likely comammox *Nitrospira*, colonizing virtually all oxic and micro-oxic environments. This finding was expectedly similar to that in engineered ecosystems [4]. A recent global-scale survey based on genomic profiling revealed that comammox *Nitrospira* outnumber canonical *Nitrospira* in various freshwater and soil environments [14], highlighting the ecological success of comammox *Nitrospira* in natural ecosystems as well as engineered ecosystems. As a critical complement, this study not only demonstrated that large rivers were hotspots for comammox communities but also presented a new recognition of their considerable abundance contribution to nitrifiers in natural ecosystems on Earth.

Landforms, as a macroscopic factor controlling climates, soils and nutrients, have been demonstrated to influence bacterial communities in large rivers [26, 61]. We found that the biogeography of riverine nitrifiers was associated with landforms. Comammox *Nitrospira* were more abundant among the AOPs and *Nitrospira* genus in the plateau, mountain and foothill, which are generally characterized by a high elevation, intensive solar radiation and weak human activity. Particularly, clade B dominated the comammox community on the Qinghai-Tibetan Plateau, the world’s third pole covered by glaciers and permafrost, and was then replaced by clade A downstream. Previous studies showed that comammox clade B had higher abundances in groundwater-related ecosystems [4, 7, 60]. Comammox clade A had comparable abundances with or outcompeted clade B in five landforms from the mountain to plain, where clade-A-Ic species were generally the dominant comammox guild. The major comammox *Nitrospira* in the nonplateau landforms of the Yangtze River was quite different from those reported in sediments of the Yellow River (*Ca*. N. nitrificans of clade A-IIa) [13], the backwater region of the upper Mississippi River (*N*. *inopinata* of clade A-Ia) [22], and various engineered ecosystems (clade A-Ia, A-IIa or A-IIb) [4, 5, 9, 10]. Distinct variation in dominant sublineages within clades A-I and A-II was also observed across landforms from the plateau to plain. These findings implied broader niches of comammox clade A in natural environments, which was consistent with the finding of a previous study [13]. Differential metabolic potentials and ecophysiologies among nitrifiers [7, 11] are suspected to enable distinct biogeography in large rivers, highlighting the urgent need to reassess nitrification corresponding to landforms.

Robust distance–decay patterns of comammox *Nitrospira* and AOPs were acquired in the Yangtze River, with higher spatial turnover rates found for the planktonic communities. Similarly, the entire bacterial communities of the Yangtze River also exhibited significant distance–decay patterns [26]. Both deterministic processes of environmental selection and stochastic events of dispersal limitation (pure geographic influence) gave rise to the distance decay [62]. The relative importance of selection and stochastic processes (e.g., dispersal, drift, and speciation) [63] interacting to influence *β*-diversity could vary with habitats, thus leading to different spatial turnover rates [49]. Environmental selection could differentiate microbial compositions among sites, thus producing a significant distance–decay pattern, and a stronger selection process could steepen the slope of the distance–decay curve [49]. The distance–decay slopes for the planktonic comammox *Nitrospira* and AOPs were 1.7–1.8 times steeper than those of the benthic ones throughout the Yangtze River, which was attributed to the dominant role of selection or the differential fitness among these taxa under environmental pressure [49, 64]. Previously, variation in planktonic AOPs in a small reach (110 km) of the Yangtze River was found to be driven by stochastic processes [65]. Such discordance might be attributed to the regional-scale survey and overlook of comammox *Nitrospira*. The community dissimilarity of benthic comammox *Nitrospira* was more related to geographic distance (26.9%), nearly half of which was ascribed to pure geographic effects (13.0%). One explanation could be that benthic taxa experienced more dispersal limitation due to a longer residence time in riverine sediments than in water [26, 66]. Due to their attached-growth preference, benthic comammox *Nitrospira* might prefer to colonize closer sites, reducing the chance for passive dispersal from one landform to another. Generally, 11.7–21.9% of the variation was explained by the joint effects of geographic distance and environmental factors, indicating spatial dependence of environmental selection to some extent. Thus, both geographic and environmental factors, partially reflecting fundamental ecological processes [55], could be used to interpret the habitat-specific biogeography of comammox *Nitrospira* and AOPs in large rivers.

Nutrient conditions shaped niche preferences among comammox *Nitrospira* and canonical nitrifiers in the Yangtze River. The oligotrophic preference of the comammox community in the plateau/mountain/foothill regions was identified. The water therein was characterized by limited nitrogen (monitoring data: NH_3_–N, 0.07 ± 0.05 mg/L; TN, 1.14 ± 0.39 mg/L), and the sediments featured scarce carbon (TOC, 4.26 ± 3.49 g/kg) and phosphorus (TP, 0.33 ± 0.19 g/kg). Studies on ammonia-oxidation kinetics [51] and comparative genomics [7, 11] suggested that comammox *Nitrospira* survived in nitrogen- and phosphorus-limited environments, which was consistent with our findings. The abundance ratio of comammox *Nitrospira* and AOA showed an insignificant correlation with NH_3_–N, possibly because these groups had similarly high affinities for ammonia, allowing them to survive under the nutrient-limited conditions [51]. Thus, both comammox *Nitrospira* and AOA played dominant roles in the oligotrophic upper reach. Organic carbon determined niche partitioning between not only comammox guilds but also comammox *Nitrospira* and AOB in the Yangtze River. The comammox community (especially clade B) had a greater abundance than AOB in organic carbon-deficient environments. Fowler et al. [60] reported that organic carbon significantly explained variation within the comammox community in groundwater-fed rapid sand filters. Lawson and Lücker [11] demonstrated that comammox *Nitrospira* had greater energy production and a more efficient CO_2_ fixation pathway than AOB, suggesting better adaptation of comammox *Nitrospira* to carbon-limited conditions. More evidence is needed to derive from kinetic analysis of carbon fixation and degradation by the comammox community, AOB and canonical *Nitrospira* in riverine sediments.

Other environmental factors, such as pH and temperature, also determined niche separation among comammox *Nitrospira* and canonical nitrifiers in the Yangtze River. Alkalescent conditions were identified in the water (pH: 8.12 ± 0.27) and sediments (pH: 9.18 ± 0.14) of the plateau/mountain/foothill regions, in which the comammox *Nitrospira* made greater contributions to AOP and *Nitrospira* abundances. This was different from a previous notion that comammox *Nitrospira* were the most abundant nitrifiers in acidic environments [67]. For large rivers, alkaline conditions could result in low copper and iron ion availability due to hydroxide precipitation. Comammox *Nitrospira*, rather than canonical *Nitrospira*, harbored Cu homeostasis genes (*copABCD*) and cytochrome *c* biogenesis system I, which might allow them to survive in copper- and iron-limited environments, respectively [7]. Alkaline conditions might also confer high alkaline phosphatase activity in bacteria [68], allowing them to survive phosphorus starvation. An additional alkaline phosphatase (*phoD*) was indeed found in comammox *Nitrospira* genomes compared with canonical AOP genomes [7]. Further tests, likely in vitro, are needed to reveal the functions of the above genetic variation. Additionally, planktonic comammox clade B made greater contributions to nitrifier abundances at lower temperatures (9.6 ± 0.4 °C) on the Qinghai-Tibetan Plateau. Previous studies demonstrated that clade B were more abundant at similar temperatures in groundwater wells (~10 °C) [4] and rapid sand filters (8.6–13.2 °C) [60]. Based on our observations, temperature appeared to exert selective pressure between not only two comammox clades but also comammox *Nitrospira* and AOB/NOB. Although multicollinearity is a concern in such studies, no covariation was observed among these significant environmental factors because of the small VIFs. This result suggested high confidence in the overall effects of environmental factors on the comammox *Nitrospira* and canonical nitrifiers in the Yangtze River.

Since the operation of the TGD began, its influence on riverine ecosystems has drawn great attention. The abundances of comammox *Nitrospira* and AOB were significantly lower ~450 km downstream of the TGD, where AOA played a dominant role among benthic AOPs. This might be attributed to fundamental changes in physicochemical, hydrodynamic and biological characteristics between upstream and downstream sediments of the TGD [26, 27]. The impoundment of the TGD formed a large reservoir that functioned as a sediment trap upstream, reducing the sediment supply and causing riverbed scouring and particle coarsening in the mid-lower reach [69], which could inevitably influence the microbiological niche and diversity [26, 70]. Severely coarsening sediments downstream of the TGD might not benefit the formation of biofilms, flocs and aggregates for the colonization of benthic comammox *Nitrospira* and AOB [7, 56], whereas the deposition of fine sediments upstream of the TGD could be favorable. The anammox diversity also decreased significantly in the sediment-coarsening area downstream of the TGD [27]. Archaeal species are known to participate in all major biogeochemical fluxes [71], and AOA could possess broader habitat ranges than their bacterial counterparts [72]. Therefore, AOA were more abundant with the loss of bacterial competitors in the TGD-induced coarsening sediments.

A genome-binning strategy could be implemented with a supervised and/or an unsupervised method. In a large river such as the Yangtze River [26], the highly diverse microbial communities would limit the performance of solely unsupervised binning, and supervised binning based on similarity searches before unsupervised binning could provide much safer and more effective classifications for specific organisms with multiple reference genomes at the species level, though microbial dark matter might be missed to some extent [73, 74]. Furthermore, microbial communities could be affected by many biotic and abiotic factors in complex rivers. Compared with the dominant bacteria, the overall comammox *Nitrospira* made up an extremely small proportion (0.035–0.233%) of the total microbes in some samples taken from the plateau and mountainous areas (Supplementary Fig. S7), which partially explained why comammox clade-B MAGs were not recovered despite their greater relative abundance than clade A in these areas. In contrast, the larger proportion of comammox *Nitrospira* (2.9%) among the overall benthic microbes in estuarial areas (XLJ station) strongly suggested a greater opportunity for successful assembly of comammox *Nitrospira* MAGs therein. Our study confirmed the above claim by revealing the high percentage (77.9%) of benthic AOP *amoA* reads assigned to comammox *Nitrospira* (Fig. 2a) and reconstructing three MAGs (clades A-Ib, A-Ic and A-IIa, see Table 1) at XLJ. Compared with previous PCR-based observations in similar estuarial environments [13, 15], metagenomic shotgun sequencing could reduce PCR and other amplification-based cloning biases with less amplification and more starting DNA [73–75], in addition to the advantages in effective binning of comammox *Nitrospira*. Additionally, an elevated sequencing depth is always expected to have a higher chance of assembling MAGs of rare species such as comammox clade B, advancing the understanding of their ecology and evolution in complex natural environments. More efforts will be needed to complete comammox *Nitrospira* phylogenies and explore their biological relevance, even to obtain insights into comammox activity via metatranscriptomics or ecophysiological experiments in large river ecosystems.

## Conclusions

Based on the improved phylogenetic resolution achieved by assembling ten novel MAGs, we provided the first biogeography of planktonic and benthic comammox *Nitrospira* in the Yangtze River over a 6030 km continuum. Our study revealed the wide existence of comammox *Nitrospira* and their significant contributions to nitrifier abundances, constituting 30% and 46% of AOPs and displaying 30.4- and 17.9-fold greater abundances than canonical *Nitrospira* representatives in water and sediments, respectively. Comammox *Nitrospira* were found to be at greater abundances (34–87% of AOPs) in typical oligotrophic environments with a higher pH and lower temperature, particularly in the plateau (clade B), mountain and foothill (clade A) areas of the upper reach. Environmental selection determined the niche replacement of planktonic comammox *Nitrospira* by canonical AOB and *Nitrospira* sublineages I/II from upstream to downstream, leading to a higher spatial turnover rate than observed for the benthic counterpart, while the dissimilarity of benthic comammox *Nitrospira* was moderately driven by geographic distance. A considerable decrease (83%) in benthic comammox *Nitrospira* abundance occurred immediately downstream of the TGD, which was consistent with a substantial decrease in the overall bacterial taxa in sediments. These findings highlight the necessity of identifying the role of complete nitrification in the biogeochemical cycles of large rivers worldwide.

## Supplementary information

Supplementary Information

## Data Availability

All the raw metagenome datasets are in the NCBI Sequence Read Archive under accession numbers SRR9924753-SRR9924814. The retrieved comammox *Nitrospira* MAGs have been deposited in NCBI GenBank under accession numbers JABMDD000000000–JABMDM000000000.
