# Supplementary material for: Comammox Nitrospira within the Yangtze River continuum: community, biogeography, and ecological drivers
Source: ISME J. 2020 Jun 18;14(10):2488–504. doi: 10.1038/s41396-020-0701-8 (PMC7490378; doi:10.1038/s41396-020-0701-8)
Supplement: Supplementary file 1 — Supplementary Information [file 41396_2020_701_MOESM1_ESM.docx]

**Supplementary Information**

**Comammox *Nitrospira* within the Yangtze River continuum: community, biogeography and ecological drivers**

**Authors**: Shufeng Liu^1,2^*, Haiying Wang^1^*, Liming Chen^1^, Jiawen Wang^1^, Maosheng Zheng^3^, Sitong Liu^1^, Qian Chen^1,4^, Jinren Ni^1,2,4^**^†^**

**Author affiliations**:

^1^College of Environmental Sciences and Engineering, Peking University; The Key Laboratory of Water and Sediment Sciences, Ministry of Education, Beijing 100871, China

^2^Beijing Innovation Center for Engineering Science and Advanced Technology, Peking University, Beijing 100871, China

^3^Key Laboratory of Regional Energy Systems Optimization, Resources and Environmental Research Academy, North China Electric Power University, Beijing 102206, China

^4^State Key Laboratory of Plateau Ecology and Agriculture, Qinghai University, Xining 810016, China

***These authors contributed equally to this work**

**^†^Corresponding author:** Jinren Ni

Postal address: Peking University, No. 5 Yiheyuan Road, Beijing 100871, China

Telephone number: +86-10-62751185

E-mail address: jinrenni@pku.edu.cn

**Competing interests statement**

The authors declare no competing financial interests.

**Contents:**

**SI Materials and Methods**

Acquisition of spatial and environmental data

DNA extraction and metagenomic shotgun sequencing

Construction of the *amoA* amino acid sequence database

Hybrid annotation pipeline used for short-read screening

Scaffold taxonomic assignments

Manual inspections of the recovered comammox *Nitrospira* MAGs

Functional annotation and metabolic pathway reconstruction

Phylogenetic tree construction for the key ammonia oxidation genes

Phylogenetic analysis of the comammox and canonical *Nitrospira* genomes

**SI Figures and tables**

**Figure S1** Relative abundances of the top 20 most abundant prokaryotic phyla and genera in the water and sediments at each sampling site.

**Figure S2** Genome comparisons between comammox *Nitrospira* *inopinata* (the only complete nitrifier isolated to date) and other available *Nitrospira* spp., including 10 novel comammox *Nitrospira* MAGs reconstructed in the present study.

**Figure S3** Maximum-likelihood trees of the comammox-related *amoA*, *amoB*, *amoC* and concatenated *amoAB* amino acid sequences.

**Figure S4** Gene schematics of the key ammonia oxidation pathway in the 10 novel comammox *Nitrospira* MAGs reconstructed from the Yangtze River.

**Figure S5** Phylogenetic analysis of the comammox and canonical *Nitrospira* genomes.

**Figure S6** Pairwise ANI values of the 36 *Nitrospira* genomes or concatenated EMGs.

**Figure S7** Relative abundances of comammox *Nitrospira* among all microbes in the water and sediments at each sampling site.

**Figure S8** Pearson’s correlations between numbers of comammox *Nitrospira* reads obtained by utilizing the *amoA* gene and 37 EMGs in each hydrologic site.

**Figure S9** NMDS diagrams showing the compositional differences of comammox *Nitrospira*, AOA and AOB among different sample groups obtained from 27 sites along the Yangtze River.

**Figure S10** Comparisons of the relative abundances of comammox *Nitrospira*, AOA and AOB between the water and sediment samples.

**Figure S11** Pearson’s correlations between the significant environmental factors (selected in the dbRDA) and abundance ratios of comammox clades A and B.

**Figure S12** Spatial dependencies of the significant environmental factors (selected in the dbRDA).

**Table S1** General information for the 27 sampling sites along the Yangtze River.

**Table S2** Statistics of raw reads and clean reads in each metagenomic dataset.

**Table S3** General descriptions of the 26 previously reported comammox and canonical *Nitrospira* genomes used in the present study.

**Table S4** Discovery or isolation sources of the 26 previously reported comammox and canonical *Nitrospira* genomes used in this study.

**Table S5** Average relative abundances of the top 10 most abundant AOA and AOB taxa in water samples corresponding to different landforms.

**Table S6** Average relative abundances of the top 10 most abundant AOA and AOB taxa in sediment samples corresponding to different landforms.

**Table S7** Summary statistics of distance-decay curves for comammox *Nitrospira* and AOP communities characterized by the curvilinear distance in specific habitats.

**Table S8** Envfit results of the dbRDA for relationships between planktonic comammox *Nitrospira* and AOP community Bray-Curtis dissimilarity and geographic and environmental variables.

**Table S9** Envfit results of the dbRDA for relationships between benthic comammox *Nitrospira* and AOP community Bray-Curtis dissimilarity and geographic and environmental variables.

**Table S10** Detailed information for each dominant AOP taxon with a significant difference (*p* < 0.05) in relative abundance between sediments collected immediately upstream and downstream of the TGD.

**SI Materials and Methods**

**Acquisition of spatial and environmental data**

The geographic coordinates of sampling sites were recorded by a handheld GPS device (Magellan, USA). The curvilinear distances between pairwise sites were calculated using ArcGIS software (v 10.3). We then generated a geographic distance matrix corresponding to the sites. The landforms that the Yangtze River flows through were retrieved from Liu et al. [1] and Wang et al. [2] and further refined by publicly available sources. Environmental factors including temperature, pH, dissolved oxygen (DO), chemical oxygen demand (COD), total nitrogen (TN), nitrate (NO_3_-N), ammonia (NH_3_-N) and total phosphorus (TP) for all the water samples were determined according to the issued protocols of Environmental Quality Standards for Surface Water (GB3838-2002) (http://kjs.mee.gov.cn/hjbhbz/bzwb/shjbh/shjzlbz/) (Ministry of Ecology and Environment of China). Sediment pH, total organic carbon (TOC), TN, NO_3_-N and NH_3_-N and TP contents were measured as described by Zhu et al. [3]. All the analyses were performed in triplicate. The obtained environmental factors were then normalized using min-max standardization.

**DNA extraction and metagenomic shotgun sequencing**

Total genomic DNA of each sample was extracted multiple times utilizing the FastDNA^®^ SPIN Kit for Soil (MP Biomedicals, USA) following the manufacturer’s instructions. Replicated DNA extracts of individual samples were mixed together for DNA quality evaluations by a NanoDrop ND-2000 instrument (Thermo Fisher Scientific, USA). Metagenomic sequencing data of a few sediment samples were not acquired due to sampling difficulties or because the DNA amount (< 1 µg) and concentration (< 10 ng µL^-1^) were not sufficient for paired-end library construction. Finally, a total of 62 metagenomic sequencing datasets were obtained for 30 water and 32 sediment samples from the Yangtze River.

**Construction of the *amoA* amino acid sequence database**

A set of *amoA* amino acid sequences of comammox *Nitrospira*, AOA and AOB were collected from the NCBI protein database by searching the key words “ammonia monooxygenase subunit A” and “*amoA*” [4, 5]. Redundant sequences were dereplicated by using CD-HIT with a 100% identity over a 100% alignment length [6]. The remaining nonredundant (NR) *amoA* amino acid sequences were aligned using MAFFT (v 7.310) [7], followed by phylogenetic tree construction using FastTree (v 2.1) with 1 000 bootstrap replicates [8], where a Jones-Taylor-Thornton evolutionary model [9, 10] and CAT approximation with 20 rate categories were employed. With considerations of both NCBI taxonomies and phylogenetic characteristics, manual curation of the NR *amoA* sequence set was carried out. After genome binning, the *amoA* sequences identified on the comammox *Nitrospira* MAGs retrieved from the Yangtze River were supplemented to form the final reference sequence pool.

**Hybrid annotation pipeline used for short-read screening**

A hybrid annotation pipeline was employed to screen the clean reads against *amoA* sequence sets with a rapid speed and an ideal accuracy [11]. Briefly, UBLAST (one of the tools in USEARCH) (v 7.0.1090) [12] was first used to screen the clean reads against *amoA* sequence sets with an *e*-value < 10^-5^ and “-accel” of 0.5, and the potential matched reads were then subjected to BLASTX (v 2.2.31+) against the above sequence sets with an *e*-value < 10^-5^ and “-max_target_seqs” of 1. Only those reads with an identity ≥ 80% and alignment length ≥ 75 bp were assigned as corresponding *amoA*-like sequences. Custom bash scripts were used to extract the *amoA*-like sequences for further analysis.

**Scaffold taxonomic assignments**

We used the criteria suggested by Ma et al. [13] to assign specific microbial taxonomies to the assembled scaffolds. MetaGeneMark (v 3.38) [14] was first applied to find open reading frames (ORFs) on all the scaffolds. Then, the predicted amino acid ORFs were queried against the NCBI NR database using DIAMOND (v 0.8.38) [15] with the parameters -e 1e-5 -f 6 -k 1, with the follow-up analysis in MEGAN (v 6.11.1) using the lowest common ancestor algorithm to identify taxonomies of the ORFs [16]. If ≥ 50% of the ORFs on a scaffold were attributed to comammox *Nitrospira* genomes, the scaffold was preliminarily assigned that taxonomy. The composite comammox *Nitrospira* scaffolds were extracted from each assembly using a custom bash script.

**Manual inspections of the recovered comammox *Nitrospira* MAGs**

First, candidate contaminants were identified by GC-coverage plots of all the scaffolds in each comammox *Nitrospira* MAG, and those outlying scaffolds were removed from each MAG due to divergent GC contents or inconsistent coverages [5]. Subsequently, a total of 107 essential single-copy genes in each comammox *Nitrospira* MAG were identified by hidden Markov models according to Albertsen et al [17]. Among the scaffolds having multiple (that is, more than one) duplicates of essential single-copy genes, the shorter scaffolds were removed from the MAG. Furthermore, redundant single-copy genes identified by CheckM (v 1.0.7) [18] were considered during the inspections of comammox *Nitrospira* MAGs. Similar to the above process, among the scaffolds having multiple redundant CheckM single-copy genes, the shorter scaffolds were removed. The longest discarded scaffold was 7.2 kbp in length. After manual curation, the average contamination of comammox *Nitrospira* MAGs decreased from 23.4% to 3.2%.

**Functional annotation and metabolic pathway reconstruction**

The amino acid sequences of predicted ORFs in the available comammox and canonical *Nitrospira* genomes were queried against the Clusters of Orthologous Groups (COG) [19] and Kyoto Encyclopedia of Genes and Genomes (KEGG) [20] databases using DIAMOND (v 0.8.38) [15] with the parameters -e 1e-5 -f 6 -k 1. Metabolic pathways were inferred based on KEGG pathway maps (https://www.kegg.jp/kegg/pathway.html).

**Phylogenetic tree construction for the key ammonia oxidation genes**

The available *amoA*, *amoB* and *amoC* amino acid sequences of all the comammox *Nitrospira* genomes were identified based on the functional annotation results. The above *amoA* and *amoB* sequences identified in the individual genomes were also concatenated. Some outgroup sequences were downloaded from the NCBI protein database. Sequences were aligned using MAFFT (v 7.310) [7], and poorly aligned regions (positions missing from 90% or more of the total aligned sequences) were removed using TrimAl (v 1.3) [21]. The sequences with very short lengths or poor alignment qualities were excluded to avoid bias. Maximum-likelihood trees were constructed using FastTree (v 2.1) with 1 000 bootstrap replicates [8], where a Jones-Taylor-Thornton evolutionary model [9, 10] and CAT approximation with 20 rate categories were utilized.

**Phylogenetic analysis of the comammox and canonical *Nitrospira* genomes**

A total of 37 elite marker gene families, which have largely congruent phylogenetic histories, were identified and concatenated using PhyloSift (v 1.0.1) with the “all” command [22]. The concatenated amino acid sequences were aligned using MAFFT (v 7.310) [7], and poorly aligned regions (positions missing from 90% or more of the total aligned comammox and canonical *Nitrospira* sequences) were removed using TrimAl (v 1.3) [21]. Genomes with low alignment quality were not used for tree construction. The phylogenetic trees were constructed using 1 000 bootstraps by MEGA (v 7.0.26) [23], where a Jones-Taylor-Thornton substitution model [9, 24] with corrections for a gamma distribution (10 discrete categories) and a WAG substitution model [5, 25, 26] with a gamma distribution (5 discrete categories) with invariant sites (G + I) were employed for the neighbor-joining and maximum-likelihood trees, respectively.


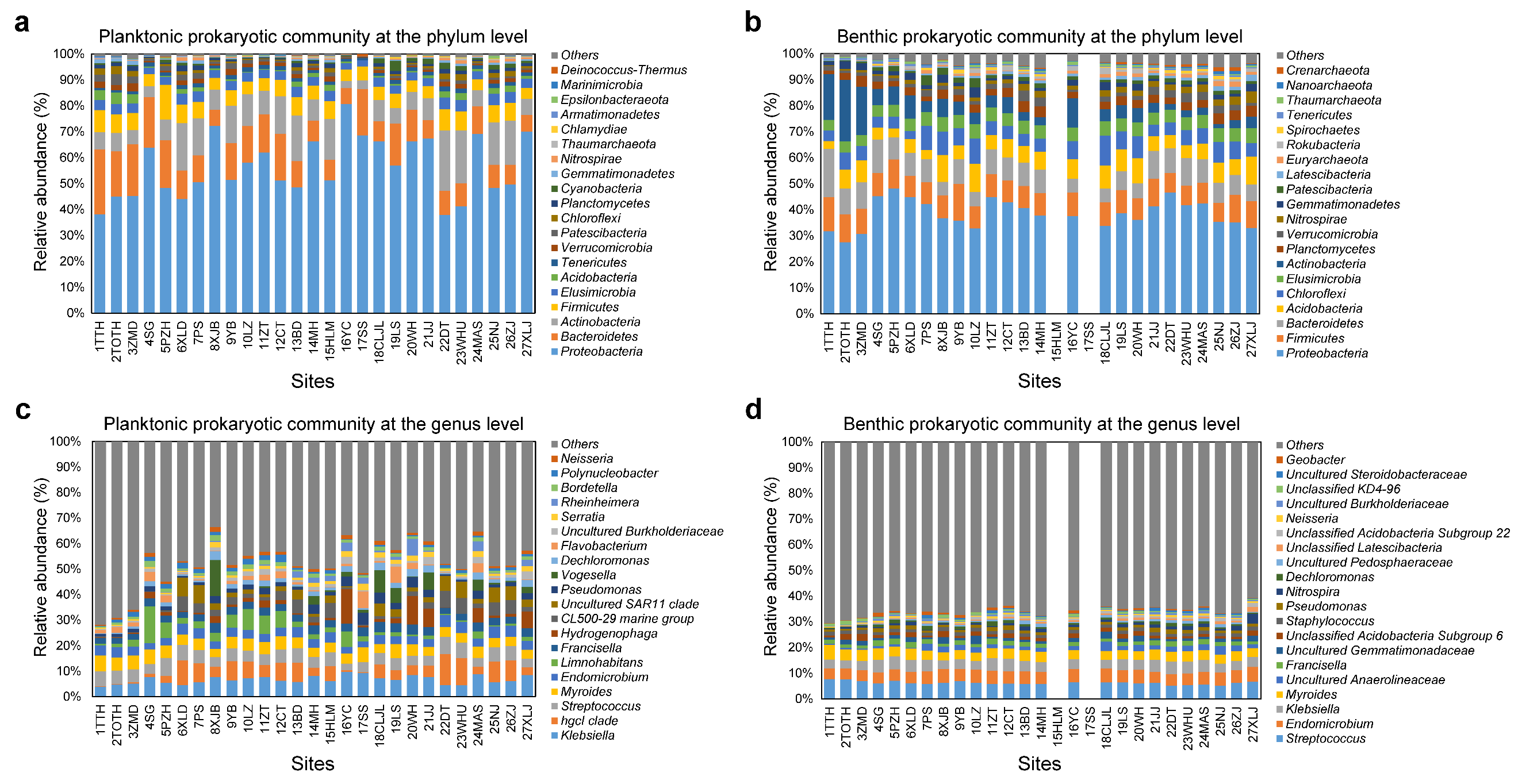


**Figure S1** Relative abundances of the top 20 most abundant prokaryotic phyla (a, b) and genera (c, d) in the water and sediments at each sampling site.


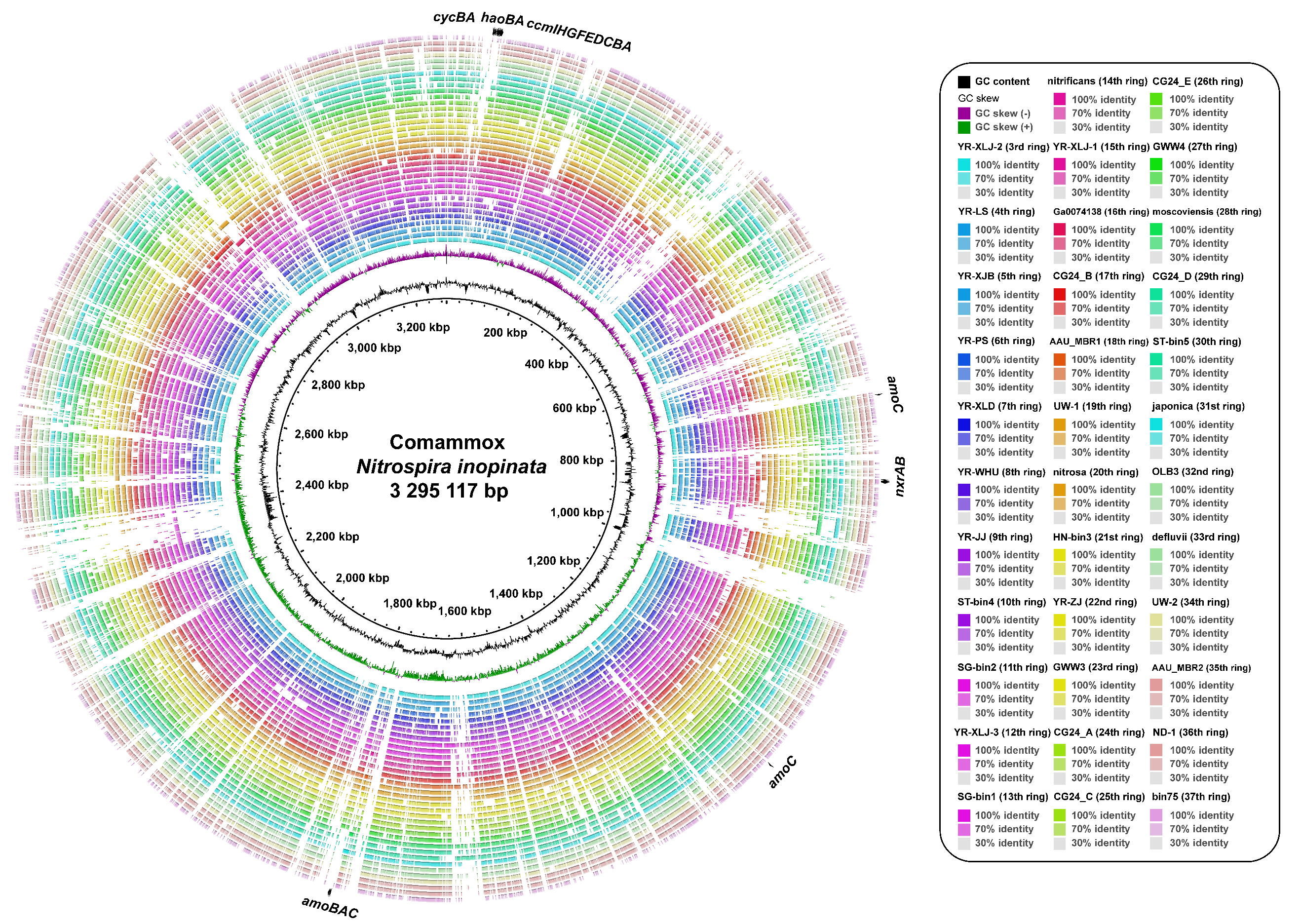


**Figure S2** Genome comparisons between comammox *Nitrospira* *inopinata* (the only complete nitrifier isolated to date [27]) and other available *Nitrospira* spp., including 10 novel comammox *Nitrospira* MAGs reconstructed in the present study. The complete genome of *Nitrospira* *inopinata* was chosen as the reference. The two innermost rings show GC content and skew, respectively. The corresponding key ammonia oxidation- and nitrite oxidation-related genes are presented in the outermost ring in black. Genome BLAST results are displayed in the remaining rings (from the 3rd to 37th).


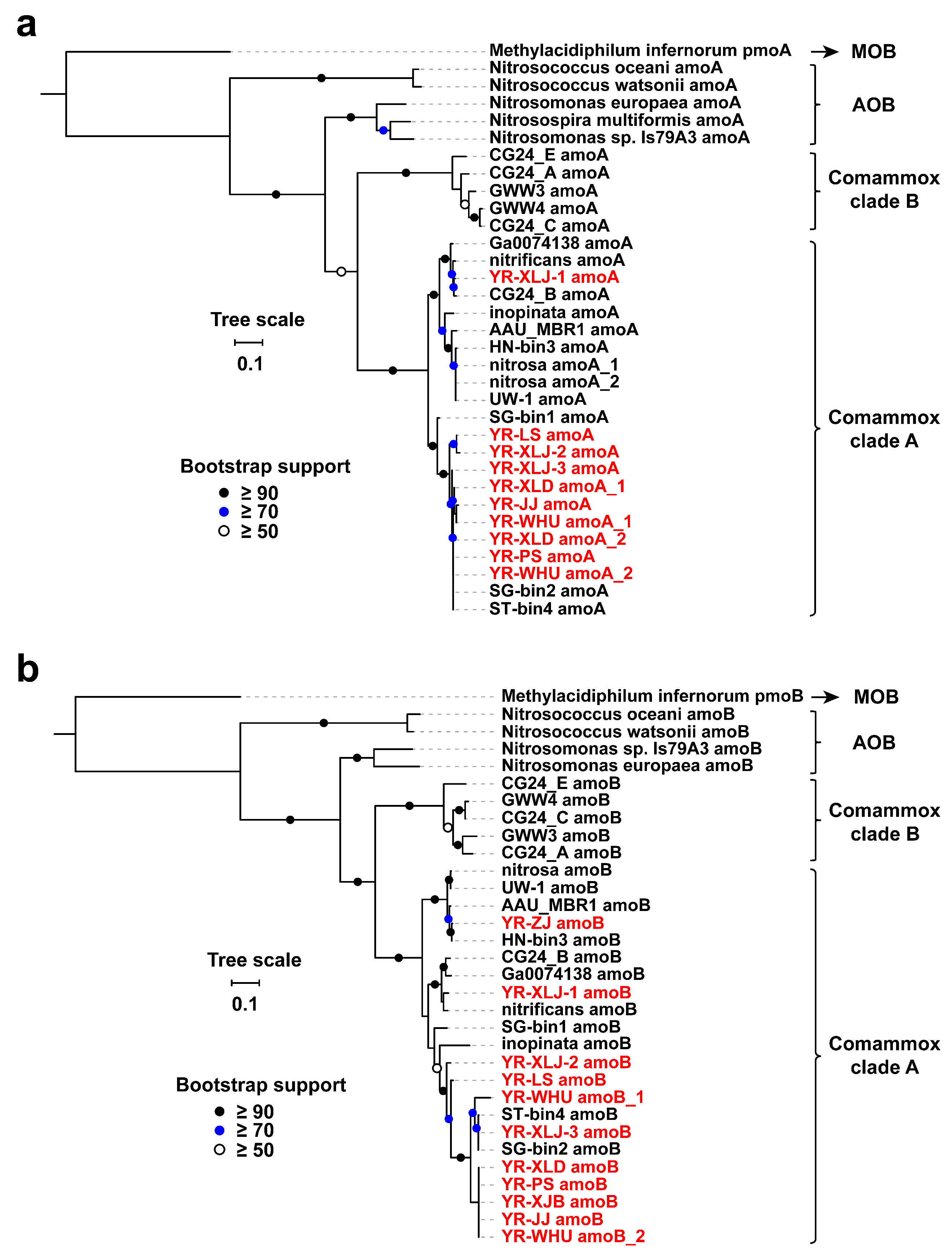


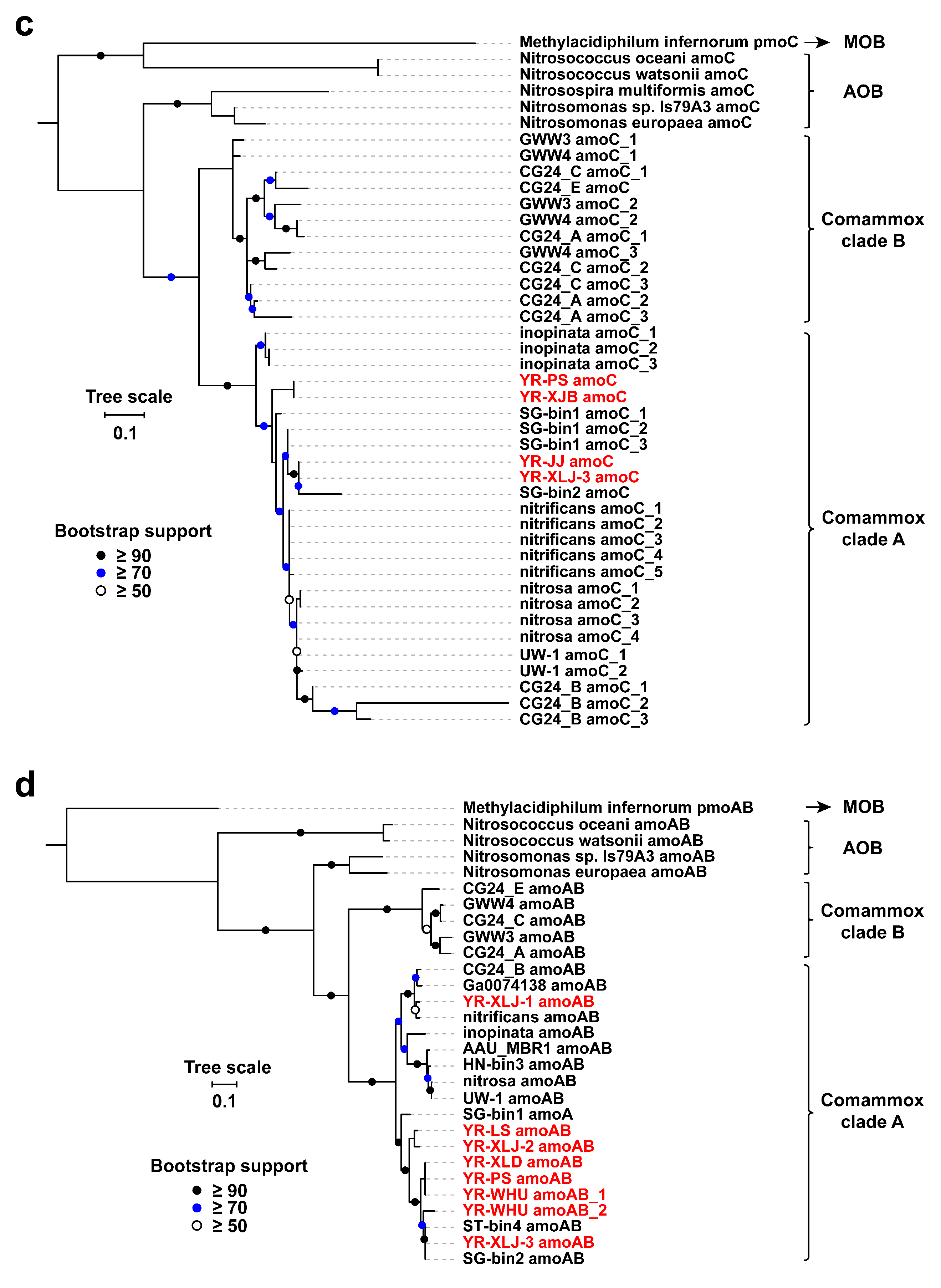


**Figure S3** Maximum-likelihood trees of the comammox-related *amoA* (a), *amoB* (b), *amoC* (c) and concatenated *amoAB* (d) amino acid sequences. The available *amo* sequences identified in the retrieved comammox *Nitrospira* MAGs in the present study (red color) and previously published comammox *Nitrospira* genomes associated with some outgroup sequences (AOB, ammonia-oxidizing bacteria; MOB, methane-oxidizing bacteria) were used for tree construction. Bootstrap values ≥ 50 are indicated with circles on the branches.


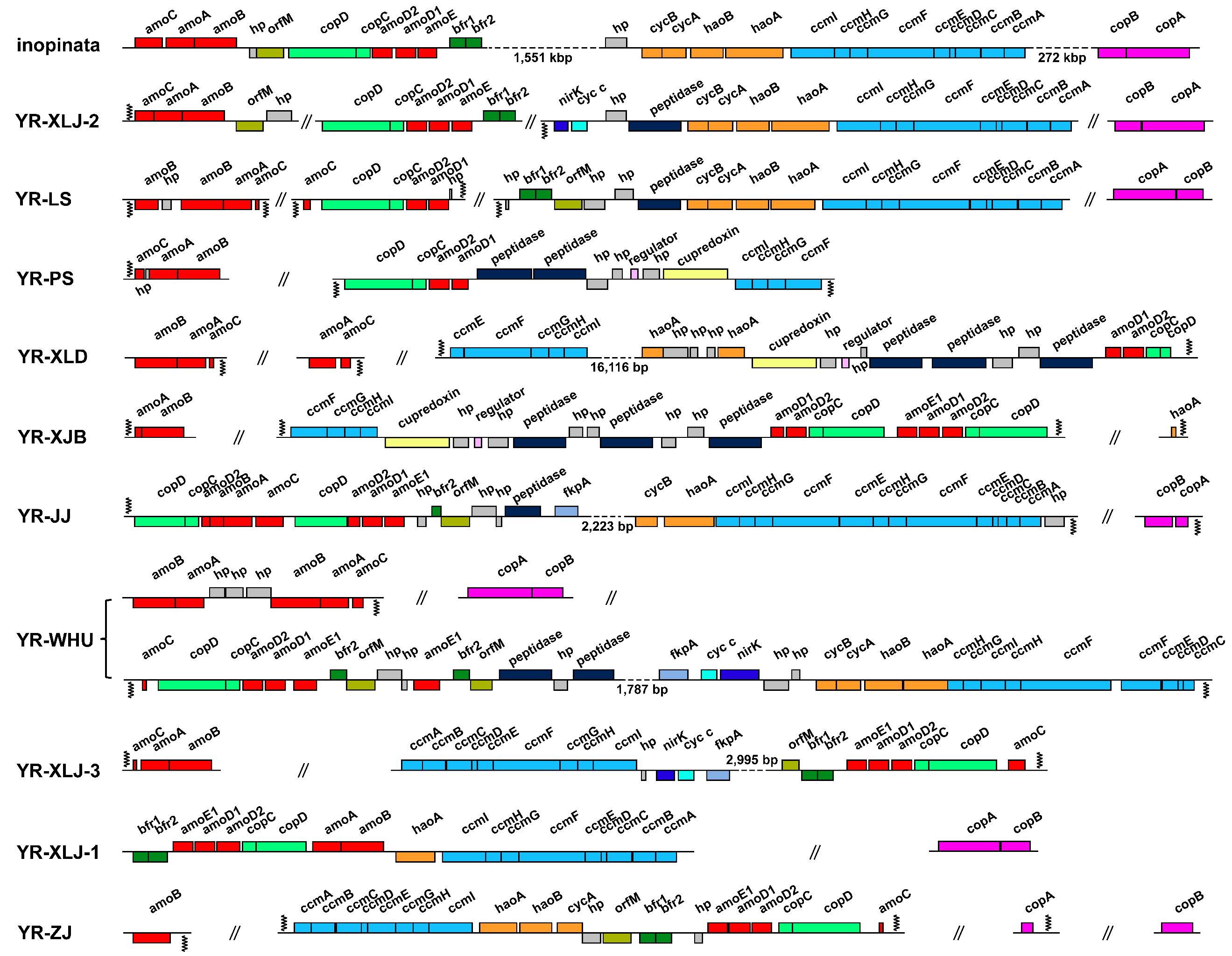


**Figure S4** Gene schematics of the key ammonia oxidation pathway in the 10 novel comammox *Nitrospira* MAGs reconstructed from the Yangtze River. Functions of the encoded genes (or gene operons) are indicated by colors. Hypothetical proteins (hp) are labeled in gray. Dotted black lines suggest continuity in locus organization where genes of little interest are not exhibited. Parallel double lines designate a break possibly due to metagenomic scaffold fragmentation. Wiggly lines show the ends of scaffolds. Genes and noncoding regions are drawn to scale. Upper and lower blocks denote forward and reverse coding strands, respectively.


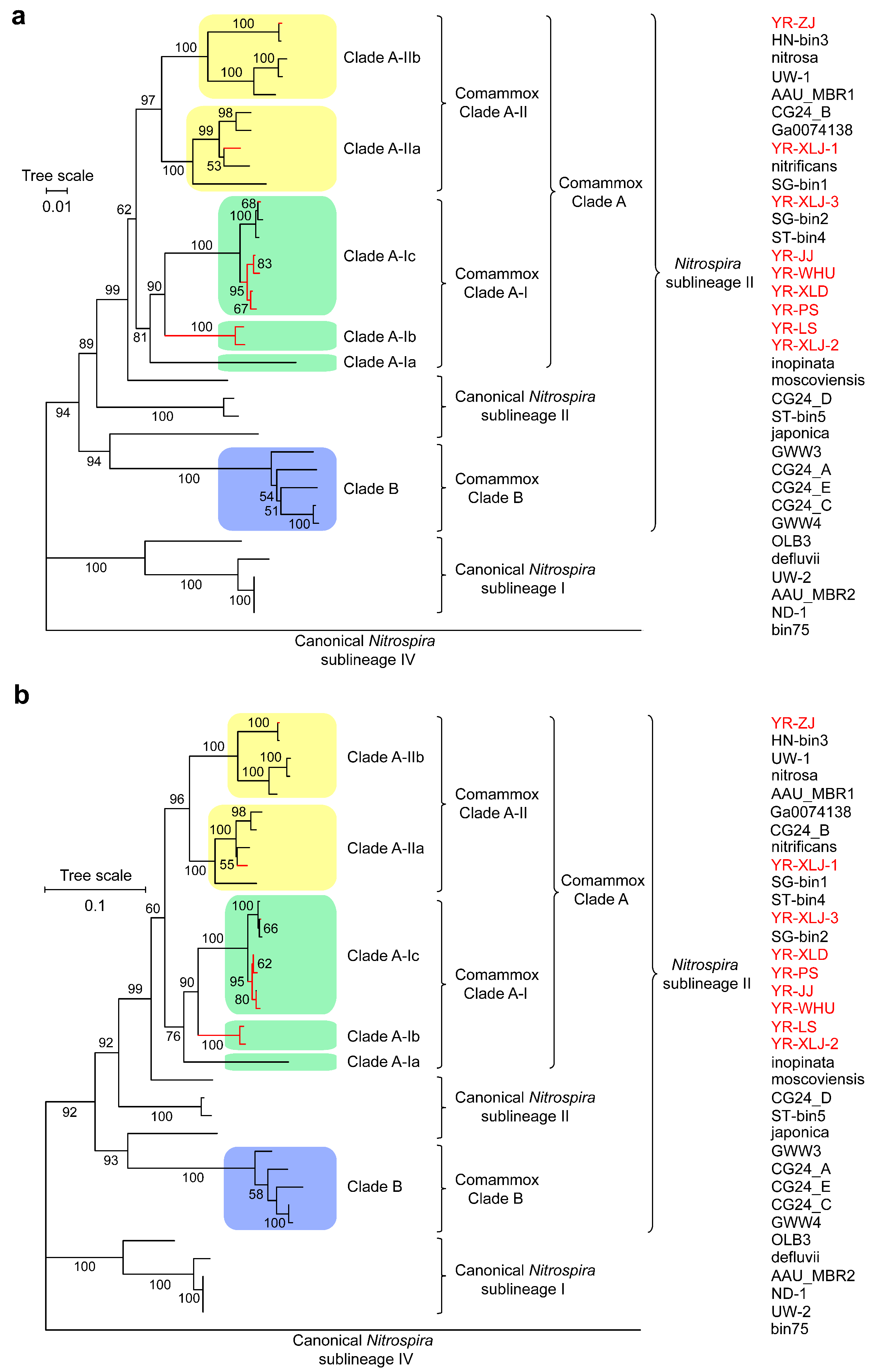


**Figure S5** Phylogenetic analysis of the comammox and canonical *Nitrospira* genomes. Both a neighbor-joining tree (a) and a maximum-likelihood tree (b) were constructed based on the concatenated alignment of 37 EMGs that were identified in all 36 *Nitrospira* genomes, including 26 previously published genomes (Supplementary Tables S3 and S4) and 10 comammox *Nitrospira* MAGs retrieved in this study. The YR-XJB bin was excluded from tree construction due to poor alignment quality. Bootstrap values ≥ 50 are indicated on the branches. The comammox *Nitrospira* MAGs retrieved from the Yangtze River are labeled in red.


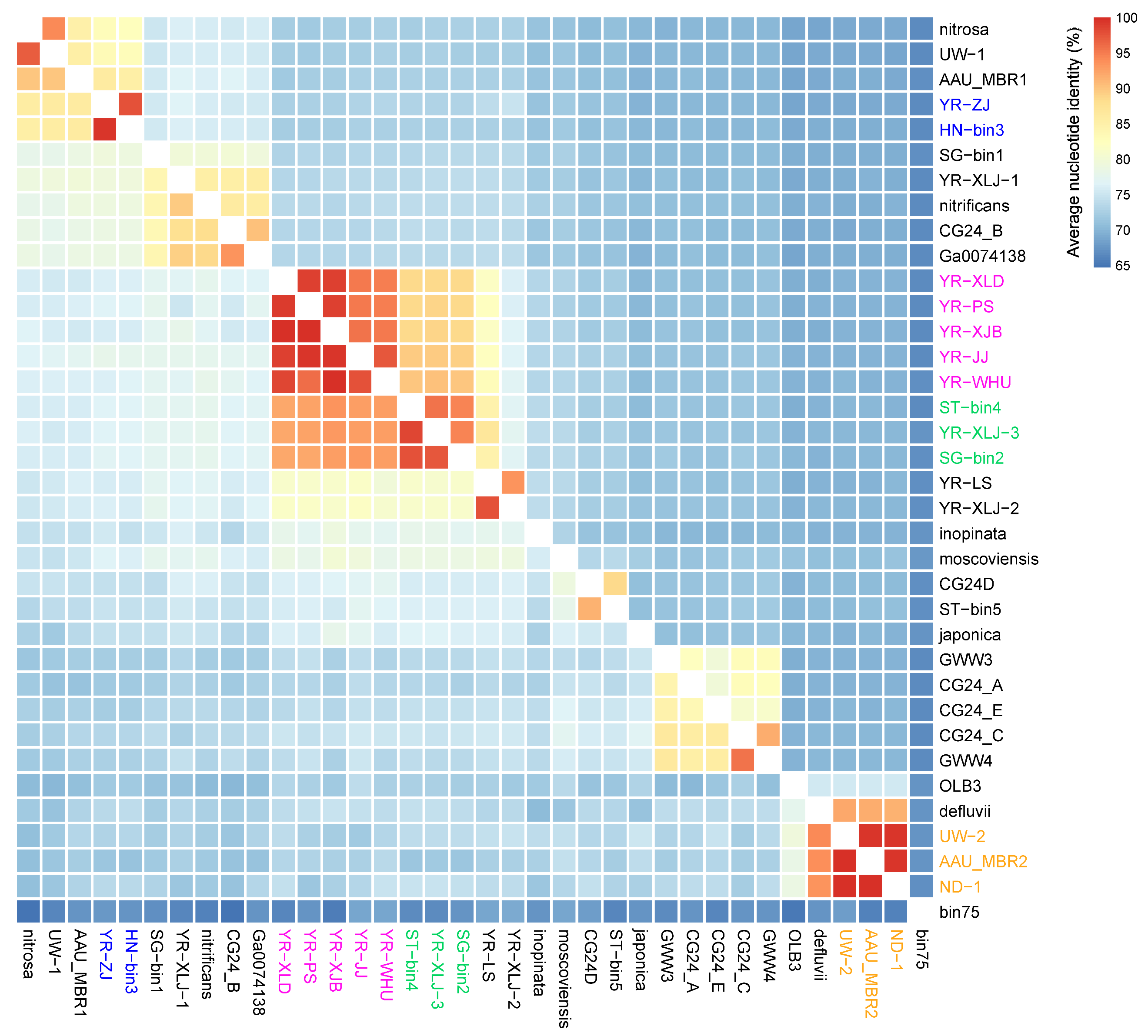


**Figure S6** Pairwise ANI values of the 36 *Nitrospira* genomes (upper triangular matrix) or concatenated EMGs (lower triangular matrix). Based on the species level cut-off of an ~95% genome-wide ANI [28], multiple genomes belonging to one species are labeled in blue, purple, green and yellow, respectively. Other genomes (in black) represent individual *Nitrospira* species.





**Figure S7** Relative abundances of comammox *Nitrospira* among all microbes in the water (a) and sediments (b) at each sampling site. All the relative abundance metrics used are based on the normalized reads mapped to the whole genomes of comammox *Nitrospira*.


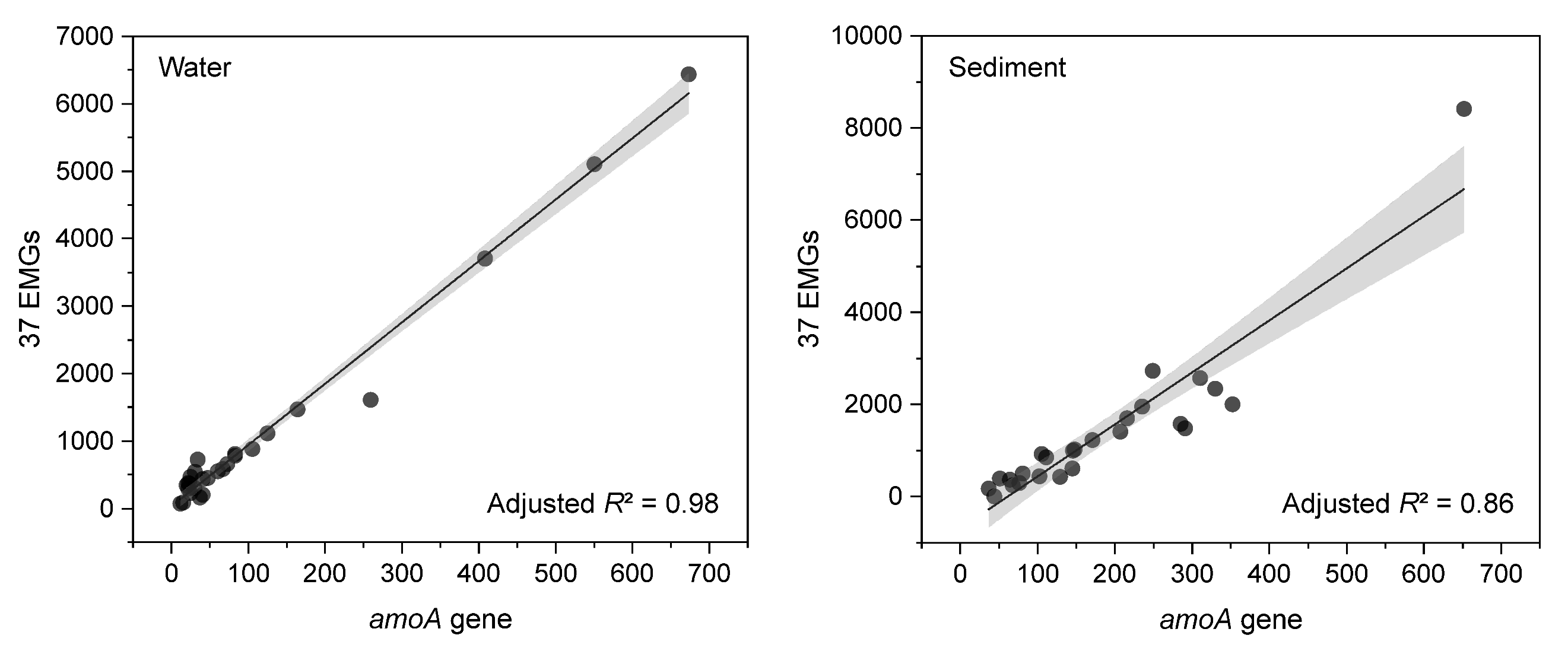


**Figure S8** Pearson’s correlations between numbers of comammox *Nitrospira* reads obtained by utilizing the *amoA* gene and 37 EMGs at each hydrologic site. Black lines indicate the ordinary least square linear regressions, with the shaded area representing 95% confidence intervals. The adjusted *R*^2^ values are provided.


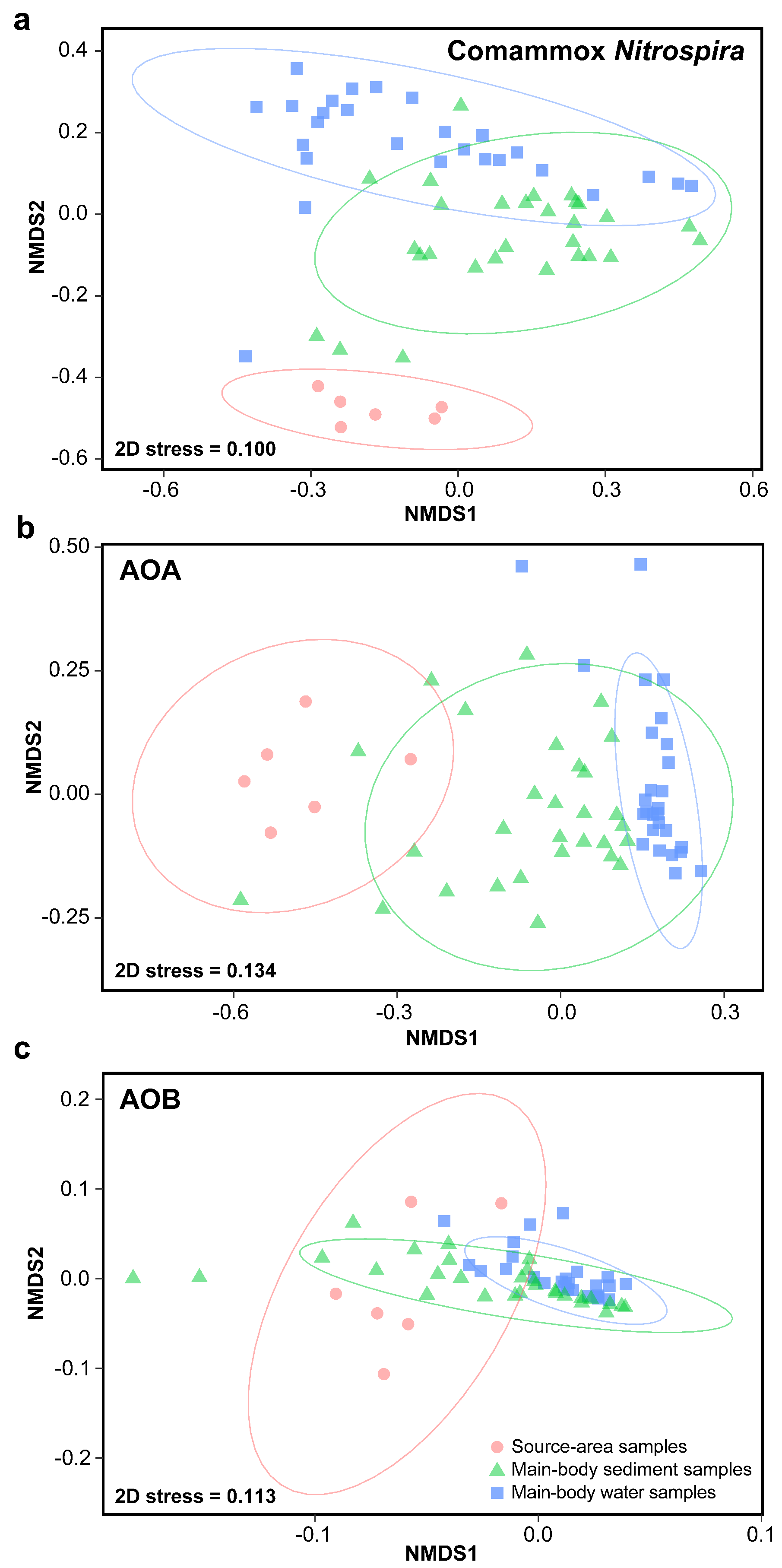


**Figure S****9** NMDS diagrams showing the compositional difference of comammox *Nitrospira* (a), AOA (b) and AOB (c) among different sample groups obtained from 27 sites along the Yangtze River. The 95% ellipses illustrate distinct sampling groups. All the relative abundance metrics used are based on the normalized *amoA* gene hits.

**
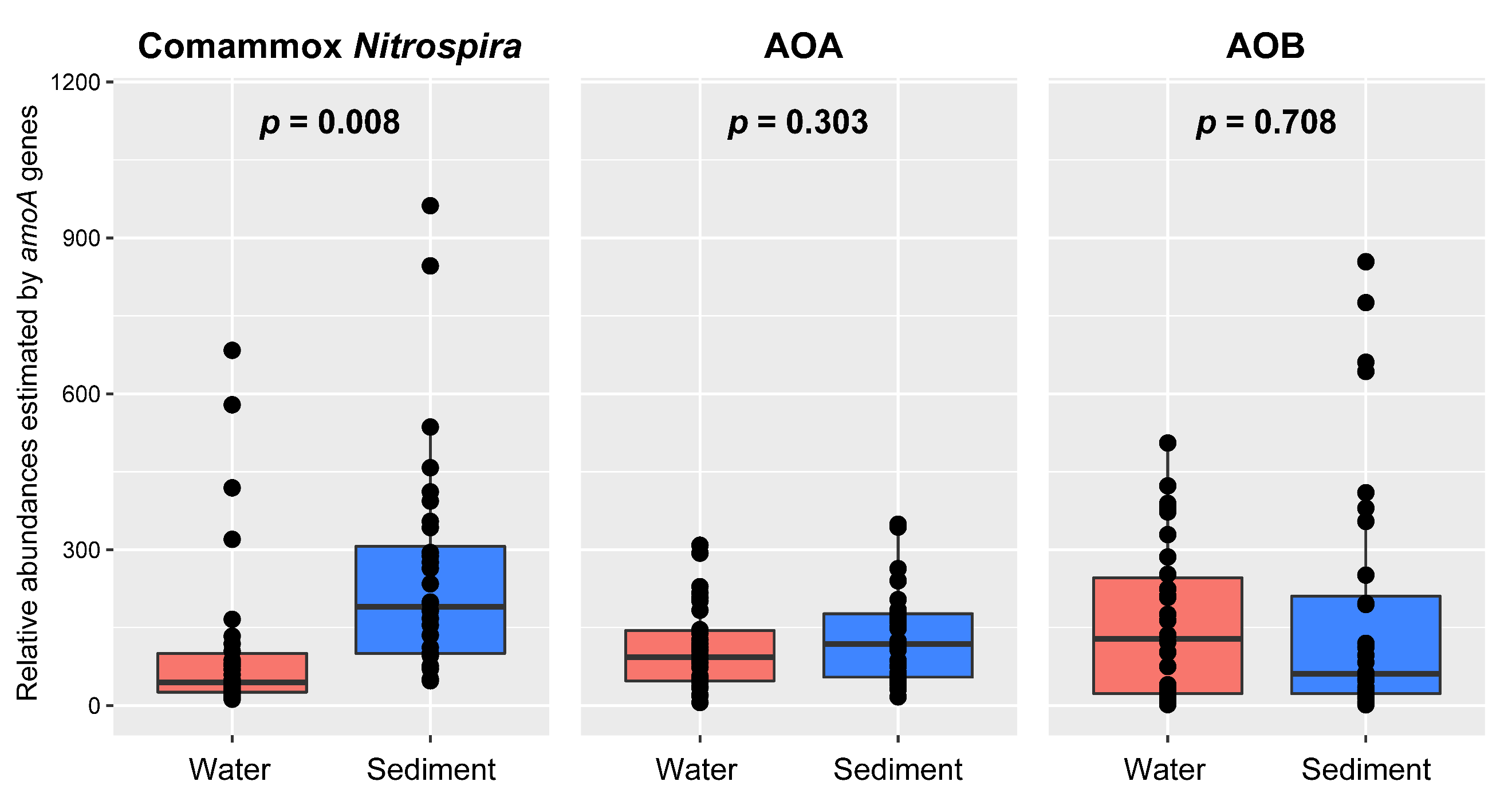
**

**Figure S10** Comparisons of the relative abundances of comammox *Nitrospira*, AOA and AOB between the water and sediment group samples. The *p* values of one-way ANOVA are provided. All the relative abundance metrics used are based on the normalized *amoA* gene hits.


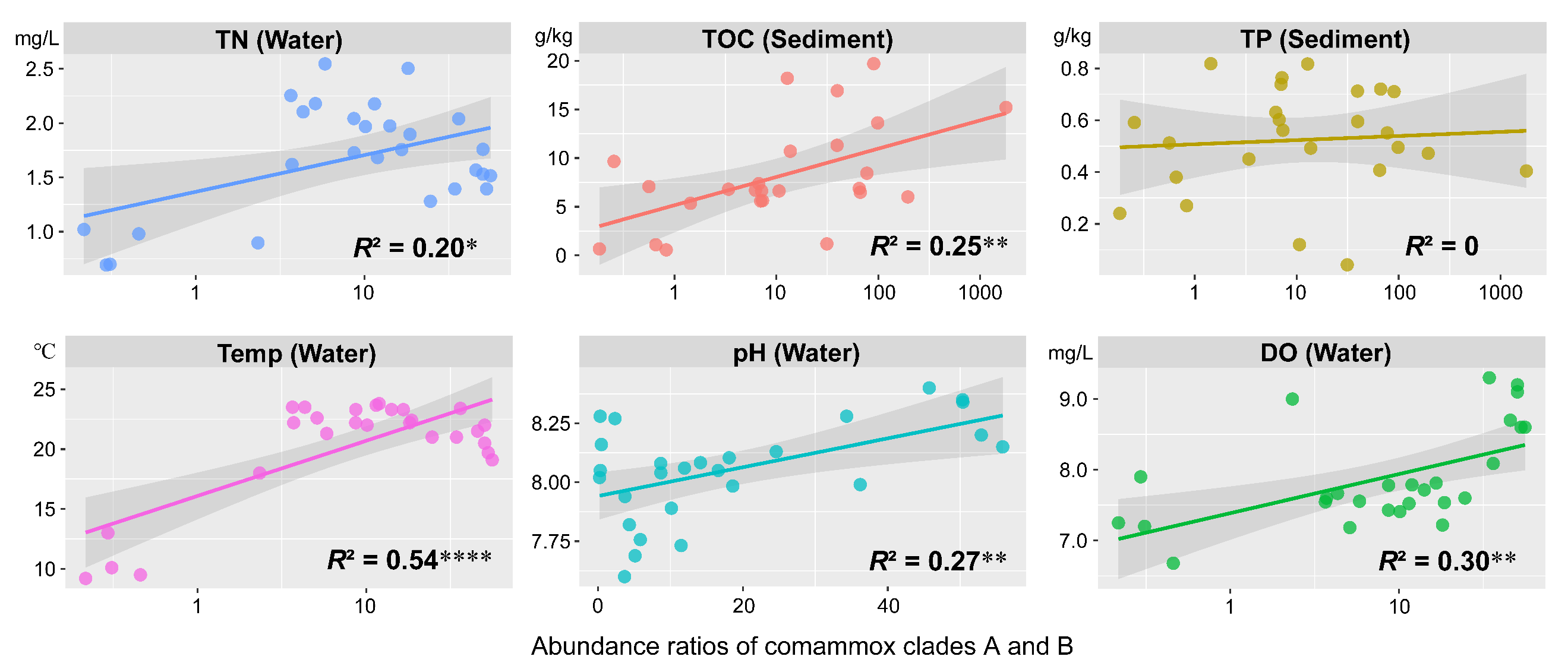


**Figure S11** Pearson’s correlations between the significant environmental factors (selected in the dbRDA) and abundance ratios of comammox clades A and B. Colored lines indicate the ordinary least square linear regressions, with the shaded area representing 95% confidence intervals. The adjusted *R*^2^ values are provided. Asterisks denote the significance of correlations (*****p* = 0.0001, ***0.0001 < *p* < 0.001, **0.001 < *p* < 0.01 and *0.01 < *p* < 0.05). TN, total nitrogen; TOC, total organic carbon; TP, total phosphorus; Temp, temperature; DO, dissolved oxygen. The abundance ratios of comammox clades A and B are based on the normalized reads mapped to 37 EMGs.


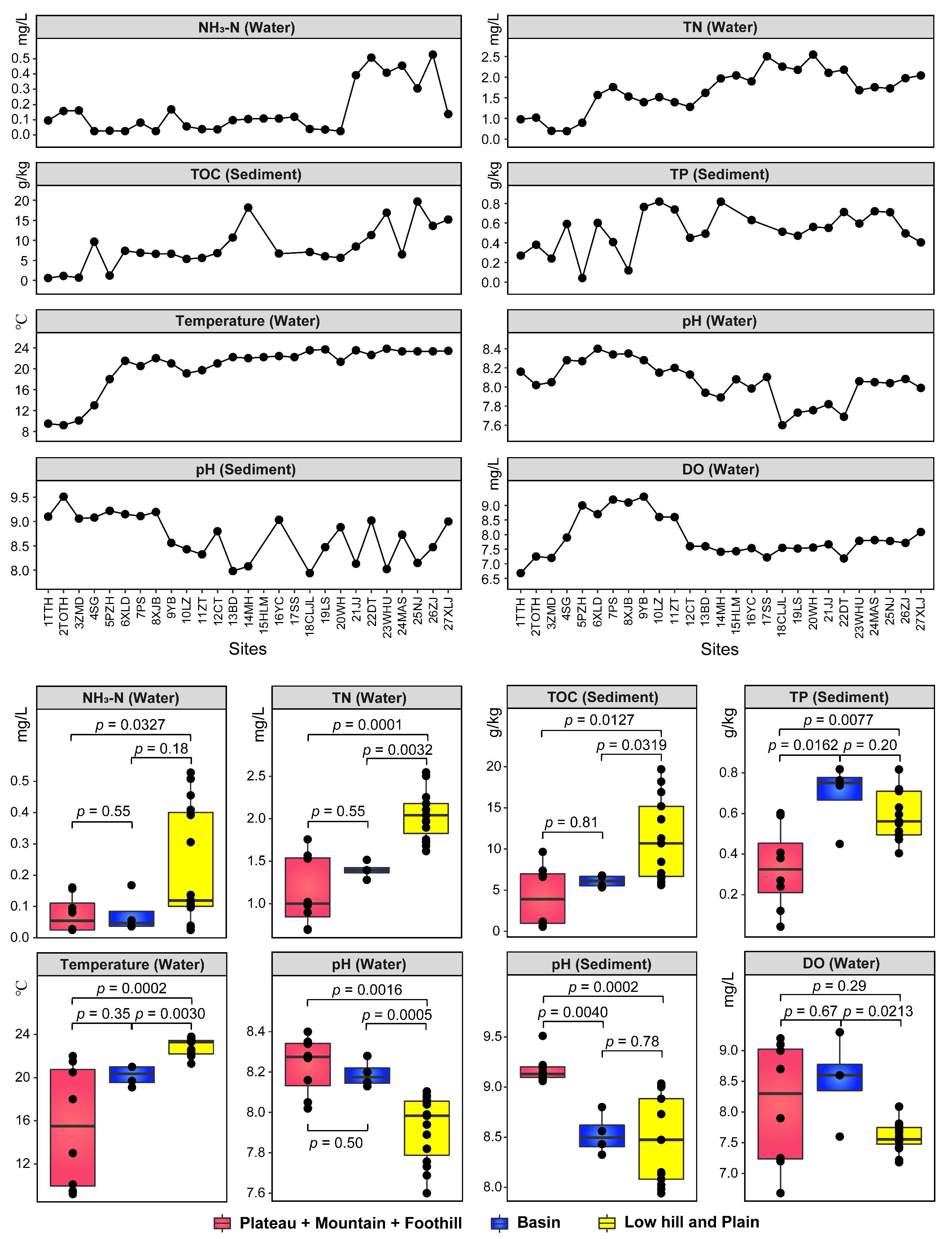


**Figure S12** Spatial dependencies of the significant environmental factors (selected in the dbRDA). Line charts indicate how these environmental factors vary from site to site along the Yangtze River. Boxplots indicate the significant differences in these environmental factors between distinct landforms along the Yangtze River. The *p* values of pairwise Mann-Whitney tests are provided.

**Table S1** General information for the 27 sampling sites along the Yangtze River.

| Site No. | Full name | Abbreviated name | Longitude (°E)^a^ | Latitude (°N)^a^ | Curvilinear distance to the estuary (km) | Obtained metagenomic datasets^b^ |
| --- | --- | --- | --- | --- | --- | --- |
| 1 | Tuotuohe | TTH | 92.44 | 34.22 | 6 027 | W/S |
| 2 | Tongtianhe | TOTH | 93.03 | 34.15 | 5 946 | W/S |
| 3 | Zhimenda | ZMD | 97.24 | 33.01 | 5 203 | W/S |
| 4 | Shigu | SG | 99.98 | 26.88 | 4 299 | W/S |
| 5 | Panzhihua | PZH | 101.70 | 26.57 | 3 706 | W/S |
| 6 | Xiluodu | XLD | 103.66 | 28.25 | 2 931 | W/S |
| 7 | Pingshan | PS | 104.17 | 28.65 | 2 811 | W/S |
| 8 | Xiangjiaba | XJB | 104.38 | 28.65 | 2 783 | W/S |
| 9 | Yibin | YB | 104.65 | 28.77 | 2 747 | W/S |
| 10 | Luzhou | LZ | 105.55 | 28.90 | 2 612 | W/S |
| 11 | Zhutuo | ZT | 105.85 | 29.02 | 2 521 | W/S |
| 12 | Cuntan | CT | 106.60 | 29.62 | 2 382 | W/S |
| 13 | Badong | BD | 110.40 | 31.04 | 1 852 | W/S |
| 14 | Miaohe | MH | 110.90 | 30.88 | 1 795 | W/S |
| 15 | Huanglingmiao | HLM | 111.12 | 30.85 | 1 771 | W |
| 16 | Yichang | YC | 111.28 | 30.69 | 1 737 | W/S |
| 17 | Shashi | SS | 112.26 | 30.29 | 1 577 | W |
| 18 | Chenglingjilian | CLJL | 113.15 | 29.45 | 1 333 | W/S |
| 19 | Luoshan | LS | 113.32 | 29.67 | 1 303 | W/S |
| 20 | Wuhan | WH | 114.32 | 30.62 | 1 104 | W/S |
| 21 | Jiujiang | JJ | 116.00 | 29.74 | 863 | W/S |
| 22 | Datong | DT | 117.64 | 30.78 | 632 | W/S |
| 23 | Wuhu | WHU | 118.34 | 31.46 | 500 | W/S |
| 24 | Maanshan | MAS | 118.47 | 31.77 | 456 | W/S |
| 25 | Nanjing | NJ | 118.94 | 32.17 | 382 | W/S |
| 26 | Zhenjiang | ZJ | 119.66 | 32.18 | 309 | W/S |
| 27 | Xuliujing | XLJ | 120.96 | 31.77 | 171 | W/S |

^a^The longitude and latitude of each site were determined by GPS.

^b^Obtained metagenomic datasets: W/S, raw metagenomic sequences were acquired from both water and sediment samples at this site; W, raw metagenomic sequences were obtained from only water samples at this site.

**Table S2** Statistics of raw reads and clean reads in each metagenomic dataset.

| Metagenomic dataset ID | Number of raw reads | Number of bases in raw reads (10^8^) | Number of clean reads | Number of bases in clean reads (10^8^) | Percentage of clean reads (%) |
| --- | --- | --- | --- | --- | --- |
| 1TTH_W | 88 481 080 | 133.61 | 86 840 381 | 128.87 | 98.15 |
| 2TOTH_W | 79 691 884 | 120.33 | 78 002 311 | 115.69 | 97.88 |
| 3ZMD_W | 100 869 672 | 152.31 | 95 459 321 | 140.22 | 94.63 |
| 4SG_W | 111 430 208 | 168.26 | 99 345 357 | 139.00 | 89.15 |
| 5PZH_W | 115 536 372 | 174.46 | 101 682 671 | 140.91 | 88.01 |
| 6XLD_W | 95 010 178 | 143.47 | 90 459 230 | 128.87 | 95.21 |
| 7PS_W | 98 474 550 | 148.70 | 96 585 959 | 139.30 | 98.08 |
| 8XJB_W | 97 415 056 | 147.10 | 95 243 507 | 136.90 | 97.77 |
| 9YB_W | 98 941 372 | 149.40 | 97 007 313 | 140.11 | 98.05 |
| 10LZ_W | 94 826 672 | 143.19 | 92 864 273 | 134.05 | 97.93 |
| 11ZT_W | 108 314 826 | 163.56 | 106 242 418 | 154.50 | 98.09 |
| 12CT_W | 94 544 242 | 142.76 | 92 815 379 | 134.81 | 98.17 |
| 13BD_W1 | 129 695 962 | 195.84 | 127 405 849 | 184.64 | 98.23 |
| 13BD_W2 | 119 094 752 | 179.83 | 116 986 106 | 169.61 | 98.23 |
| 14MH_W | 115 099 940 | 173.80 | 112 962 867 | 163.81 | 98.14 |
| 15HLM_W1 | 95 752 370 | 144.59 | 93 548 625 | 134.85 | 97.70 |
| 15HLM_W2 | 82 643 564 | 124.79 | 81 138 150 | 117.97 | 98.18 |
| 16YC_W1 | 82 255 676 | 124.21 | 80 588 788 | 116.89 | 97.97 |
| 16YC_W2 | 83 536 768 | 126.14 | 81 671 706 | 118.25 | 97.77 |
| 17SS_W | 99 533 278 | 150.30 | 97 608 834 | 141.87 | 98.07 |
| 18CLJL_W | 86 429 048 | 130.51 | 84 831 289 | 123.33 | 98.15 |
| 19LS_W | 83 000 020 | 125.33 | 81 441 773 | 118.23 | 98.12 |
| 20WH_W | 93 284 714 | 140.86 | 91 568 558 | 133.11 | 98.16 |
| 21JJ_W | 86 594 574 | 130.76 | 85 075 120 | 123.99 | 98.25 |
| 22DT_W | 75 479 422 | 113.97 | 74 447 950 | 108.92 | 98.63 |
| 23WHU_W | 69 913 812 | 105.57 | 68 837 100 | 100.48 | 98.46 |
| 24MAS_W | 88 457 174 | 133.57 | 87 297 833 | 127.90 | 98.69 |
| 25NJ_W | 82 800 920 | 125.03 | 81 595 240 | 119.28 | 98.54 |
| 26ZJ_W | 93 818 690 | 141.67 | 92 426 096 | 134.94 | 98.52 |
| 27XLJ_W | 80 969 224 | 122.26 | 79 706 971 | 114.83 | 98.44 |
| 1TTH_S | 102 651 634 | 155.00 | 101 616 593 | 152.34 | 98.99 |
| 2TOTH_S | 107 937 722 | 162.99 | 106 645 695 | 159.53 | 98.80 |
| 3ZMD_S | 85 909 930 | 129.72 | 84 793 226 | 126.91 | 98.70 |
| 4SG_S | 77 823 896 | 117.51 | 76 266 622 | 112.36 | 98.00 |
| 5PZH_S | 107 750 012 | 161.63 | 106 376 460 | 157.56 | 98.73 |

**Table S2** Continued.

| Metagenomic dataset ID | Number of raw reads | Number of bases in raw reads (10^8^) | Number of clean reads | Number of bases in clean reads (10^8^) | Percentage of clean reads (%) |
| --- | --- | --- | --- | --- | --- |
| 6XLD_S | 102 779 336 | 155.20 | 101 348 489 | 148.94 | 98.61 |
| 7PS_S | 96 506 220 | 145.72 | 95 184 809 | 139.89 | 98.63 |
| 8XJB_S | 73 549 420 | 111.06 | 71 901 926 | 105.79 | 97.76 |
| 9YB_S | 65 245 302 | 98.52 | 63 605 908 | 92.17 | 97.49 |
| 10LZ_S | 92 526 356 | 139.71 | 89 989 236 | 130.48 | 97.26 |
| 11ZT_S | 68 215 920 | 103.01 | 66 674 220 | 97.90 | 97.74 |
| 12CT_S | 77 029 730 | 116.31 | 74 840 073 | 109.28 | 97.16 |
| 13BD_S1 | 70 593 518 | 106.60 | 69 114 034 | 100.63 | 97.90 |
| 13BD_S2 | 69 732 612 | 105.30 | 68 474 589 | 101.06 | 98.20 |
| 14MH_S1 | 79 776 238 | 120.46 | 76 260 000 | 109.28 | 95.59 |
| 14MH_S2 | 78 475 770 | 118.50 | 76 806 685 | 112.49 | 97.87 |
| 14MH_S3 | 72 761 002 | 109.87 | 71 302 056 | 105.02 | 97.99 |
| 16YC_S | 101 506 996 | 153.28 | 98 783 534 | 143.51 | 97.32 |
| 18CLJL_S1 | 76 526 074 | 115.55 | 74 343 461 | 108.26 | 97.15 |
| 18CLJL_S2 | 73 228 508 | 110.58 | 71 586 940 | 105.08 | 97.76 |
| 19LS_S | 106 321 714 | 159.48 | 105 028 392 | 155.60 | 98.78 |
| 20WH_S | 84 394 648 | 127.44 | 81 733 360 | 118.11 | 96.85 |
| 21JJ_S1 | 80 211 926 | 121.12 | 77 753 577 | 112.48 | 96.94 |
| 21JJ_S2 | 84 878 512 | 128.17 | 83 129 342 | 122.41 | 97.94 |
| 22DT_S | 81 066 170 | 122.41 | 78 872 432 | 113.97 | 97.29 |
| 23WHU_S1 | 90 509 448 | 136.67 | 88 870 561 | 129.77 | 98.19 |
| 23WHU_S2 | 75 938 882 | 114.67 | 74 147 228 | 108.20 | 97.64 |
| 24MAS_S | 81 842 548 | 123.58 | 80 088 636 | 116.48 | 97.86 |
| 25NJ_S | 61 035 236 | 92.16 | 59 447 032 | 87.00 | 97.40 |
| 26ZJ_S1 | 75 762 564 | 114.40 | 74 188 608 | 107.93 | 97.92 |
| 26ZJ_S2 | 64 181 094 | 96.91 | 62 574 967 | 91.39 | 97.50 |
| 27XLJ_S | 67 784 632 | 102.35 | 65 779 555 | 95.65 | 97.04 |

**Table S3** General descriptions of the 26 previously reported comammox and canonical *Nitrospira* genomes used in the present study.

| Genome ID | Size (Mbp) | GC content (%) | Completeness (%) | Contamination (%) | Accession | References |
| --- | --- | --- | --- | --- | --- | --- |
| nitrosa^a^ | 4.4 | 54.8 | 96.8 | 2.3 | CZQA00000000^f^ | [29] |
| UW-1^a^ | 3.9 | 54.9 | 95.8 | 3.6 | NIUT00000000^f^ | [30] |
| AAU_MBR1^a^ | 3.5 | 55.0 | 86.3 | 2.7 | Mmgenome server^g^ | [4] |
| HN-bin3^a^ | 2.9 | 55.8 | 78.2 | 1.4 | LVWU00000000^f^ | [5] |
| SG-bin1^a^ | 4.4 | 56.1 | 95.9 | 3.7 | LVWS00000000^f^ | [5] |
| nitrificans^a^ | 4.1 | 56.6 | 96.8 | 2.7 | NZ_CZPZ00000000^f^ | [29] |
| CG24_B^a^ | 3.2 | 55.2 | 92.2 | 4.7 | NEWS00000000^f^ | [31] |
| Ga0074138^a^ | 4.1 | 55.1 | 87.7 | 2.8 | LNDU00000000^f^ | [32] |
| ST-bin4^a^ | 2.9 | 56.8 | 93.0 | 4.6 | MSXM00000000^f^ | [5] |
| SG-bin2^a^ | 3.7 | 56.8 | 95.9 | 3.7 | LVWT00000000^f^ | [5] |
| inopinata^a^ | 3.3 | 59.2 | 96.8 | 4.8 | NZ_LN885086^f^ | [4] |
| moscoviensis^b^ | 4.6 | 62.0 | 95.9 | 6.6 | CP011801^f^ | [33] |
| CG24_D^b^ | 3.4 | 57.8 | 92.2 | 3.2 | NEWQ00000000^f^ | [31] |
| ST-bin5^b^ | 4.0 | 58.0 | 91.8 | 5.7 | MSXN00000000^f^ | [5] |
| japonica^b^ | 4.1 | 59.0 | 96.8 | 3.9 | LT828648^f^ | [34] |
| GWW3^c^ | 3.5 | 55.8 | 92.2 | 3.2 | Mmgenome server^g^ | [4] |
| CG24_A^c^ | 3.6 | 55.7 | 87.2 | 4.1 | NEWT00000000^f^ | [31] |
| CG24_E^c^ | 3.5 | 55.7 | 93.1 | 3.2 | NEWP00000000^f^ | [31] |
| CG24_C^c^ | 3.0 | 56.1 | 93.1 | 2.7 | NEWR00000000^f^ | [31] |
| GWW4^c^ | 3.0 | 55.9 | 84.0 | 1.8 | Mmgenome server^g^ | [4] |
| OLB3^d^ | 3.8 | 60.4 | 91.0 | 4.3 | JZQY00000000^f^ | [35] |
| defluvii^d^ | 4.3 | 59.0 | 97.7 | 2.3 | FP929003^f^ | [36] |
| UW-2^d^ | 3.5 | 59.2 | 94.4 | 4.7 | NIUU00000000^f^ | [30] |
| AAU_MBR2^d^ | 4.3 | 59.1 | 96.8 | 5.5 | Mmgenome server^g^ | [4] |
| ND-1^d^ | 4.5 | 58.9 | 97.7 | 2.7 | FWEX00000000^f^ | [37] |
| bin75^e^ | 3.0 | 56.2 | 94.1 | 2.7 | MPMZ00000000^f^ | [38] |

^a^Belonging to comammox clade A (*Nitrospira* sublineage II).

^b^Belonging to canonical *Nitrospira* sublineage II.

^c^Belonging to comammox clade B (*Nitrospira* sublineage II).

^d^Belonging to canonical *Nitrospira* sublineage I.

^e^Belonging to canonical *Nitrospira* sublineage IV according to the *nxrB* phylogeny (data not shown here).

^f^The NCBI accessions.

^g^The mmgenome server: http://madsalbertsen.github.io/mmgenome/.

**Table S4** Discovery or isolation sources of the 26 previously reported comammox and canonical *Nitrospira* genomes used in this study. The references are listed in Table S3.

| Genome ID | Ecosystem type | Country |
| --- | --- | --- |
| nitrosa | Recirculation aquaculture system biofilter | Netherlands |
| UW-1 | Biological nutrients removal system | USA |
| AAU_MBR1 | Pilot-scale membrane bioreactor of a conventional full-scale wastewater treatment plant | Denmark |
| HN-bin3 | Tap water | China |
| SG-bin1 | Tap water | Singapore |
| nitrificans | Recirculation aquaculture system biofilter | Netherlands |
| CG24_B | Rapid sand filter in groundwater purification | Denmark |
| Ga0074138 | Active filters at a drinking water system | USA |
| ST-bin4 | Tap water | USA |
| SG-bin2 | Tap water | Singapore |
| inopinata | Microbial biofilm attached on the surface of a pipe which was covered by hot water | Russia |
| moscoviensis | Iron pipe of a heating system | Russia |
| CG24_D | Rapid sand filter in groundwater purification | Denmark |
| ST-bin5 | Tap water | USA |
| japonica | Activated sludge of a wastewater treatment plant | Japan |
| GWW3 | Iron sludge from a groundwater well | Germany |
| CG24_A | Rapid sand filter in groundwater purification | Denmark |
| CG24_E | Rapid sand filter in groundwater purification | Denmark |
| CG24_C | Rapid sand filter in groundwater purification | Denmark |
| GWW4 | Iron sludge from a groundwater well | Germany |
| OLB3 | Anammox granules in a full-scale wastewater treatment plant | Netherlands |
| defluvii | Activated sludge of a municipal wastewater treatment plant | Germany |
| UW-2 | Biological nutrients removal system | USA |
| AAU_MBR2 | Pilot-scale membrane bioreactor of a conventional full-scale wastewater treatment plant | Denmark |
| ND-1 | Activated sludge of a sewage treatment plant | Japan |
| bin75 | Marine sponges in Mediterranean Sea | Near Slovenia |

**Table S5** Average relative abundances of the top 10 most abundant AOA and AOB taxa in water samples corresponding to different landforms. All the relative abundance metrics used are based on the normalized *amoA* gene hits.

| AOPs | NCBI accessions | Taxonomy^a^ | Plateau | Mountain | Foothill | Basin | Low hill | Plain |
| --- | --- | --- | --- | --- | --- | --- | --- | --- |
| AOA taxa | ABU40677.1 | *Nitrosocosmicus* | 1.08 | **0.45*** | 0 | 0 | 0 | 0 |
|  | ACZ06735.1 | *Nitrosocosmicus* | **2.00*** | 0 | 0.34 | 0 | 0 | 0 |
|  | ADK92883.1 | *Nitrosotalea* | 0.38 | 0.43 | **11.68*** | **2.81*** | **8.58*** | **6.74*** |
|  | ADM62314.1 | *Nitrosopelagicus* | 0.33 | 0 | **4.12*** | **1.53*** | **4.07*** | 2.88 |
|  | AFA54192.1 | *Nitrososphaera* | **3.25*** | **0.45*** | 0.34 | 0 | 0.13 | 0.21 |
|  | AFH88871.1 | *Nitrosarchaeum* | 0 | 0 | 1.02 | **1.01*** | 1.51 | 1.17 |
|  | AFH88881.1 | *Crenarchaeote* | **1.67*** | 0 | 0 | 0 | 0 | 0 |
|  | AFU59457.1 | *Nitrososphaera* | **2.20*** | 0 | 0 | 0 | 0 | 0 |
|  | AGH02483.1 | *Thaumarchaeote* | **1.50*** | **0.45*** | 0 | 0 | 0 | 0 |
|  | AGI37672.1 | *Crenarchaeote* | **1.96*** | 0 | 0 | 0 | 0 | 0 |
|  | AIF09479.1 | *Nitrosarchaeum* | 0.33 | **2.61*** | **17.86*** | **3.76*** | **23.51*** | **16.58*** |
|  | AJT39825.1 | *Crenarchaeote* | **1.59*** | 0 | 0 | 0 | 0 | 0 |
|  | ANA06102.1 | *Nitrosarchaeum* | 0 | **0.88*** | **9.29*** | **1.26*** | **10.20*** | **6.62*** |
|  | BAJ22403.1 | *Nitrosocosmicus* | **2.63*** | 0.43 | 0 | 0 | 0 | 0 |
|  | BAJ52041.1 | *Nitrosotenuis* | 0.66 | 0 | **4.79*** | 0.52 | **3.77*** | **3.19*** |
|  | CCH03860.1 | *Nitrosotenuis* | 0 | 0 | 0.69 | **0.53*** | 1.16 | 0.95 |
|  | CCH03932.1 | *Nitrosarchaeum* | 0 | **0.87*** | 0 | 0 | 0.26 | 0.11 |
|  | CCH03935.1 | *Nitrosarchaeum* | 0 | **0.87*** | **8.22*** | **2.31*** | **6.84*** | **4.24*** |
|  | CDN71505.1 | *Nitrosarchaeum* | 0.38 | **1.30*** | 0 | 0.23 | 0 | 0 |
|  | CDN71663.1 | *Nitrosocosmicus* | **1.46*** | 0 | 0 | 0 | 0 | 0 |
|  | CDN71698.1 | *Nitrosarchaeum* | 0 | 0 | **5.81*** | 0.48 | **5.55*** | **4.75*** |
|  | CDN71700.1 | *Nitrosarchaeum* | 0 | 0 | 2.37 | 0 | 3.32 | **3.03*** |
|  | CDN71707.1 | *Nitrosarchaeum* | 0 | **0.87*** | **9.61*** | **2.27*** | **10.78*** | **6.71*** |
|  | CDN71728.1 | *Nitrosarchaeum* | 0 | 0.43 | **5.79*** | **1.26*** | **7.13*** | **3.90*** |
|  | EGP94363.1 | *Nitrosarchaeum* | **2.03*** | **2.64*** | **25.73*** | **3.11*** | **24.42*** | **14.72*** |
| AOB taxa | AAF64447.1 | *Nitrosomonas* | 0.33 | **0.43*** | 0.35 | **1.53*** | 2.50 | 5.62 |
|  | AAL86638.1 | *Nitrosomonas* | 0 | 0 | **2.08*** | 1.30 | 5.01 | **10.76*** |
|  | AAM11610.1 | *Nitrosomonas* | **0.75*** | 0 | 0 | 0 | 0 | 0 |
|  | AAR27104.1 | *Nitrosomonas* | 0 | **0.45*** | 0 | 0 | 0 | 0.10 |
|  | ABB69924.1 | *Nitrosovibrio* | 0 | 0 | 0.34 | 0 | **9.95*** | 0.20 |
|  | ABM64729.1 | *Nitrosomonas* | **0.71*** | 0 | 0.68 | 0.25 | 2.06 | 5.78 |
|  | ABM91810.1 | *Nitrosomonas* | 0 | 0 | 0.34 | 0 | **6.19*** | 0.32 |
|  | ABN13010.1 | *Nitrosomonas* | **0.80*** | 0 | 0 | 0 | 0 | 0 |
|  | ABN13045.1 | *Nitrosospira* | 0 | **0.43*** | 0 | 0 | **10.94*** | 3.83 |
|  | ABN13101.1 | *Nitrosospira* | **0.42*** | 0 | 0 | 0 | 1.45 | 0 |
|  | ABQ10730.1 | *Nitrosomonas* | **0.42*** | **0.43*** | **1.03*** | 0.78 | 1.67 | 4.49 |
|  | ABS88746.1 | *Nitrosomonas* | 0.38 | 0 | **1.03*** | 0 | **5.32*** | 6.80 |
|  | ABW75239.1 | *Nitrosospira* | 0 | **0.43*** | 0 | 0 | 0 | 0 |
|  | ADJ19230.1 | *Nitrosospira* | 0 | 0 | **1.72*** | 0 | 4.64 | **8.19*** |
|  | ADJ38108.1 | *Nitrosomonas* | **0.66*** | 0 | 0 | 0 | 0 | 0 |
|  | ADN07631.1 | *Nitrosomonas* | 0 | 0 | **1.72*** | 0.26 | 3.66 | **6.82*** |
|  | ADY86578.1 | *Nitrosovibrio* | 0 | 0 | 0 | 0 | **6.37*** | 0.53 |
|  | AEI15961.1 | *Nitrosomonas* | 0 | 0 | 0.35 | 1.00 | 2.57 | **6.94*** |
|  | AEJ02636.1 | *Nitrosomonas* | 0.33 | **0.43*** | **2.41*** | **2.31*** | **14.01*** | **29.20*** |
|  | AEO92104.1 | *Nitrosomonas* | 0 | 0 | **2.04*** | **1.57*** | **7.40*** | **10.70*** |

^a^Taxonomic affiliations (phylum or genus level) of each AOA or AOB *amoA* gene were directly obtained from NCBI taxonomies or BLAST results against the NCBI reference protein database (best hit: e-value < 10^-5^).

*Bold fonts: relative abundances of the top 10 most abundant AOA and AOB taxa in corresponding landforms.

**Table S5** Continued.

| AOPs | NCBI accessions | Taxonomy^a^ | Plateau | Mountain | Foothill | Basin | Low hill | Plain |
| --- | --- | --- | --- | --- | --- | --- | --- | --- |
| AOB taxa | AEZ00349.1 | *Nitrosospira* | **0.80*** | 0 | 0.35 | **1.52*** | **6.29*** | 6.32 |
|  | AEZ00366.1 | *Nitrosospira* | 0 | **0.43*** | 0.34 | 0 | 0.93 | 0.33 |
|  | AFR69026.1 | *Nitrosospira* | 0 | 0 | **1.04*** | 1.24 | 2.71 | **7.94*** |
|  | AFR69043.1 | *Nitrosospira* | 0 | **0.45*** | 0 | 0 | 0 | 0 |
|  | AGG11850.1 | *Nitrosomonas* | **0.84*** | 0 | 0 | 0 | 0 | 0 |
|  | AHA48449.1 | *Nitrosospira* | 0 | 0 | **1.04*** | 0.74 | 0 | 0.62 |
|  | AHA62705.1 | *Nitrosospira* | 0 | 0 | 0 | 0 | **6.91*** | 0.30 |
|  | CAD56336.1 | *Nitrosomonas* | 0 | 0 | 0.34 | **1.31*** | 3.32 | **7.65*** |
|  | CAD92067.1 | *Nitrosomonas* | 0 | 0 | 0 | **2.07*** | 1.44 | 4.20 |
|  | CAD92075.1 | *Nitrosomonas* | 0.38 | 0 | 0 | **1.53*** | 3.77 | **10.09*** |
|  | OQW41106.1 | *Nitrosomonas* | **0.80*** | 0 | 0 | 0.53 | 0.37 | 3.58 |
|  | SEP15831.1 | *Nitrosomonas* | 0 | **0.43*** | 0 | **1.69*** | 0.58 | 3.18 |
|  | SNX58714.1 | *Nitrosomonas* | 0 | **0.43*** | **3.13*** | **2.47*** | **9.39*** | **21.09*** |
|  | SOD16174.1 | *Nitrosomonas* | 0 | 0 | 0.34 | **1.55*** | 2.10 | 4.68 |
|  | WP_090719658.1 | *Nitrosomonas* | **0.80*** | 0 | 0 | 0 | 0.64 | 0.87 |

^a^Taxonomic affiliations (phylum or genus level) of each AOA or AOB *amoA* gene were directly obtained from NCBI taxonomies or BLAST results against the NCBI reference protein database (best hit: e-value < 10^-5^).

*Bold fonts: relative abundances of the top 10 most abundant AOA and AOB taxa in corresponding landforms.

**Table S6** Average relative abundances of the top 10 most abundant AOA and AOB taxa in sediments corresponding to different landforms. All the relative abundance metrics used are based on the normalized *amoA* gene hits.

| AOPs | NCBI accessions | Taxonomy^a^ | Plateau | Mountain | Foothill | Basin | Low hill | Plain |
| --- | --- | --- | --- | --- | --- | --- | --- | --- |
| AOA taxa | ABU40686.1 | *Crenarchaeote* | **1.72*** | **1.11*** | **2.98*** | 0.65 | 0.95 | 0.81 |
|  | ABU91406.1 | *Crenarchaeote* | **1.47*** | 0 | 0.65 | 0.32 | 0.14 | 0.20 |
|  | ACZ06639.1 | *Nitrosocosmicus* | **1.98*** | 0 | 0 | 0.97 | 0.42 | 0.07 |
|  | ADK92883.1 | *Nitrosotalea* | 0 | 0 | 2.27 | **4.05*** | **5.25*** | **2.67*** |
|  | ADM62287.1 | *Nitrosopumilus* | 0 | 0.64 | 0.97 | 1.61 | **3.52*** | 1.45 |
|  | AEI16056.1 | *Nitrosocosmicus* | **1.36*** | 0 | **3.09*** | 0.97 | 0.39 | 0.80 |
|  | AEI87422.1 | *Nitrosocosmicus* | **1.25*** | **1.11*** | 0 | 0 | 0 | 0 |
|  | AFA54183.1 | *Nitrosopumilus* | 0 | 0.46 | **2.59*** | 0.37 | 0 | 1.48 |
|  | AFA54192.1 | *Nitrosocosmicus* | **1.33*** | 0.64 | 1.95 | 1.62 | 1.13 | 0.79 |
|  | AFD20481.1 | *Crenarchaeote* | 0 | **1.93*** | 0.91 | 0 | 0 | 0.17 |
|  | AFK24904.1 | *Nitrosocosmicus* | 0.31 | 0 | **3.83*** | 2.01 | 2.22 | 0.47 |
|  | AFO64496.1 | *Thaumarchaeote* | 0 | **1.28*** | 1.25 | 0.32 | 1.05 | 0.86 |
|  | AFU59457.1 | *Nitrososphaera* | **2.68*** | 0 | 1.62 | **3.41*** | **3.11*** | **2.28*** |
|  | AFX97683.1 | *Nitrosocosmicus* | **1.01*** | 0.64 | 1.45 | 0.27 | 0.86 | 0.39 |
|  | AGT79016.1 | *Nitrosopumilus* | 0 | **1.39*** | **4.08*** | 0.32 | 2.04 | 2.09 |
|  | AGU38650.1 | *Nitrosopumilus* | 0 | 0 | 0 | **2.43*** | 3.11 | 1.18 |
|  | AIE99604.1 | *Nitrosopumilus* | 0 | 0.64 | 1.30 | **7.17*** | **12.22*** | **4.00*** |
|  | AIF09479.1 | *Nitrosopumilus* | 0 | **2.03*** | **5.18*** | **11.20*** | **13.13*** | **8.65*** |
|  | AIN34939.1 | *Nitrosopumilus* | 0.39 | 0.46 | **6.16*** | 1.41 | 2.09 | 2.07 |
|  | ANA06102.1 | *Nitrosopumilus* | 0 | 0.64 | 0.32 | **2.65*** | **3.90*** | 1.41 |
|  | APW84889.1 | *Nitrosocosmicus* | 0.70 | **1.11*** | 0.32 | 0 | 0 | 0 |
|  | BAJ22392.1 | *Nitrosocosmicus* | **1.16*** | 0.46 | 0 | 0 | 0.24 | 0.39 |
|  | BAJ22403.1 | *Nitrosocosmicus* | 0.63 | 0.64 | **4.71*** | 2.26 | 1.17 | 0.97 |
|  | CCH03935.1 | *Nitrosopumilus* | 0 | 0.46 | 0.32 | 2.00 | 2.37 | **2.13*** |
|  | CDN71537.1 | *Nitrosopumilus* | 0 | 0 | 0.97 | **4.44*** | **9.16*** | **3.55*** |
|  | CDN71611.1 | *Nitrosocosmicus* | **1.64*** | **1.93*** | 0.32 | 0.38 | 0 | 0.88 |
|  | CDN71707.1 | *Nitrosopumilus* | 0 | **1.11*** | 0.32 | **2.38*** | **4.66*** | **2.41*** |
|  | CDN71720.1 | *Nitrosopumilus* | 0 | 0 | **4.02*** | 0.27 | **3.39*** | **3.51*** |
|  | CDN71728.1 | *Nitrosopumilus* | 0 | **0.93*** | 1.95 | **3.64*** | 2.81 | **2.23*** |
|  | EGP94363.1 | *Nitrosarchaeum* | 0.39 | 0.46 | **3.24*** | **6.04*** | **10.06*** | **6.89*** |
| AOB taxa | AAB38709.1 | *Nitrosospira* | 0 | **0.64*** | 0.32 | 0 | 0 | 0 |
|  | AAC31361.1 | *Nitrosomonas* | 0 | 0 | **0.69*** | 0 | 0.38 | 1.41 |
|  | AAF64112.1 | *Nitrosomonas* | **0.71*** | 0 | 0 | 0 | 0.38 | 0.32 |
|  | AAF64448.1 | *Nitrosomonas* | **0.97*** | 0 | 0.32 | 0 | 0.86 | 0.58 |
|  | AAL86638.1 | *Nitrosomonas* | 0 | **0.93*** | **1.02*** | **1.40*** | **8.27*** | **9.65*** |
|  | AAO60367.1 | *Nitrosospira* | 0 | **0.64*** | 0 | 0 | 0.33 | 0 |
|  | ABM64729.1 | *Nitrosomonas* | 0 | 0 | 0 | **1.12*** | **3.84*** | 5.94 |
|  | ABM91817.1 | *Nitrosospira* | 0 | 0 | **1.30*** | 0 | 0 | 0.12 |
|  | ABN13010.1 | *Nitrosomonas* | **0.97*** | 0 | 0 | 0 | 0 | 0 |
|  | ABN13101.1 | *Nitrosospira* | 0 | 0 | **0.97*** | 0 | 0.14 | 0.18 |
|  | ABQ10730.1 | *Nitrosomonas* | 0 | 0 | 0 | **1.40*** | 3.41 | 5.15 |
|  | ABR29039.1 | *Nitrosospira* | 0 | **0.93*** | 0 | 0 | 0.38 | 0.33 |
|  | ACF78163.1 | *Nitrosomonas* | **0.96*** | 0 | 0 | 0 | 0 | 0 |
|  | ADJ19230.1 | *Nitrosospira* | 0 | 0 | 0 | 0.37 | 3.50 | **8.21*** |
|  | ADN07631.1 | *Nitrosomonas* | 0.39 | 0 | 0 | 0 | **4.48*** | 4.84 |

^a^Taxonomic affiliations (phylum or genus level) of each AOA or AOB *amoA* gene were directly obtained from NCBI taxonomies or BLAST results against the NCBI reference protein database (best hit: e-value < 10^-5^).

*Bold fonts: relative abundances of the top 10 most abundant AOA and AOB taxa in corresponding landforms.

**Table S6** Continued.

| AOPs | NCBI accessions | Taxonomy^a^ | Plateau | Mountain | Foothill | Basin | Low hill | Plain |
| --- | --- | --- | --- | --- | --- | --- | --- | --- |
| AOB taxa | ADZ26282.1 | *Nitrosomonas* | 0 | **0.46*** | 0.32 | 0.37 | 1.21 | 1.97 |
|  | AEH20924.1 | *Nitrosomonas* | 0 | 0 | **0.69*** | **1.12*** | 0.87 | 0.67 |
|  | AEI15961.1 | *Nitrosomonas* | 0 | 0 | 0.35 | 0 | 1.52 | **6.79*** |
|  | AEJ02636.1 | *Nitrosomonas* | 0 | **0.46*** | 0.35 | **1.80*** | **17.13*** | **24.72*** |
|  | AEO92104.1 | *Nitrosomonas* | 0 | 0 | 0 | 0 | **5.87*** | **6.54*** |
|  | AFK73408.1 | *Nitrosomonas* | 0 | 0 | **0.69*** | 0 | 0.28 | 0 |
|  | AFR69026.1 | *Nitrosospira* | 0.32 | 0 | 0 | 0.75 | **4.80*** | **6.62*** |
|  | AGG11851.1 | *Nitrosospira* | 0 | 0 | **0.69*** | 0.32 | 0.38 | 0.48 |
|  | AHA92930.1 | *Nitrosospira* | **0.65*** | 0 | 0 | 0 | 0 | 0 |
|  | ASZ84488.1 | *Nitrosospira* | 0 | **0.64*** | **0.78*** | 0 | 0.47 | 0 |
|  | BAU78244.1 | *Nitrosomonas* | 0 | **0.46*** | 0 | 0.38 | 0.29 | 0.31 |
|  | CAD56336.1 | *Nitrosomonas* | 0.32 | 0 | 0 | 0 | **4.37*** | 5.09 |
|  | CAD92067.1 | *Nitrosomonas* | **0.63*** | 0 | 0.35 | 0 | 2.53 | 2.95 |
|  | CAD92075.1 | *Nitrosomonas* | **0.65*** | 0 | 0.35 | **1.53*** | **5.39*** | **9.81*** |
|  | OQW41106.1 | *Nitrosomonas* | 0 | **0.46*** | **0.69*** | 0.32 | 3.76 | **9.19*** |
|  | SCY45393.1 | *Nitrosospira* | 0.32 | 0 | 0 | **1.10*** | 0 | 1.38 |
|  | SEP15831.1 | *Nitrosomonas* | 0.39 | **0.93*** | 0.67 | **1.93*** | **4.29*** | **12.43*** |
|  | SIO41207.1 | *Nitrosomonas* | **0.65*** | 0 | 0 | 0 | 0 | 0.24 |
|  | SNX58714.1 | *Nitrosomonas* | 0 | **0.46*** | **0.69*** | **1.44*** | **7.96*** | **11.18*** |
|  | WP_090572320.1 | *Nitrosomonas* | **0.96*** | **0.46*** | 0 | 0.38 | 2.56 | 2.55 |
|  | WP_090719658.1 | *Nitrosomonas* | **0.65*** | 0 | 0.35 | **1.53*** | 0.76 | 2.74 |

^a^Taxonomic affiliations (phylum or genus level) of each AOA or AOB *amoA* gene were directly obtained from NCBI taxonomies or BLAST results against the NCBI reference protein database (best hit: e-value < 10^-5^).

*Bold fonts: relative abundances of the top 10 most abundant AOA and AOB taxa in corresponding landforms.

**Table S7** Summary statistics of distance-decay curves for comammox *Nitrospira* and AOP communities characterized by the curvilinear distance in specific habitats.

| Comammox *Nitrospira* or AOPs | River habitats | Samples | Intercept | Slope | Adjusted *R*^2^ of ordinary least squares linear regression | Average Bray-Curtis similarity | Mantel Spearman’s rank correlation coefficient (*r*) | *p* |
| --- | --- | --- | --- | --- | --- | --- | --- | --- |
| Comammox *Nitrospira* | Water | 30 | 0.949 | -0.098 | 0.39 | 0.315 | 0.64 | 0.0001 |
| AOPs | Water | 30 | 0.868 | -0.091 | 0.53 | 0.260 | 0.74 | 0.0001 |
| Comammox *Nitrospira* | Sediment | 32 | 0.738 | -0.057 | 0.16 | 0.413 | 0.47 | 0.0001 |
| AOPs | Sediment | 32 | 0.606 | -0.051 | 0.24 | 0.279 | 0.56 | 0.0001 |

**Table S8** Envfit results of the dbRDA for relationships between planktonic comammox *Nitrospira* and AOP community Bray-Curtis dissimilarity and geographic and environmental variables.

| Variables | Comammox *Nitrospira* | | |  | AOPs | | |
| --- | --- | --- | --- | --- | --- | --- | --- |
|  | CAP1 | CAP2 | *p* |  | CAP1 | CAP2 | *p* |
| **NH_3_-N** | N.S. | | |  | 0.777 | 0.630 | 0.0260 |
| **TN** | 0.906 | -0.424 | 0.0001 |  | 0.988 | 0.152 | 0.0001 |
| **pH** | 0.403 | 0.915 | 0.0039 |  | -0.105 | -0.995 | 0.0001 |
| **DO** | -0.614 | 0.789 | 0.0001 |  | -0.768 | -0.640 | 0.0001 |
| TP | N.S. | | |  | N.S. | | |
| COD | N.S. | | |  | N.S. | | |
| **Temp** | 0.993 | -0.122 | 0.0001 |  | 0.985 | -0.171 | 0.0001 |
| **Geo** | -0.984 | 0.177 | 0.0001 |  | -0.997 | -0.073 | 0.0001 |

Abbreviations: NH_3_-N, ammonia; TN, total nitrogen; DO, dissolved oxygen; TP, total phosphorus; COD, chemical oxygen demand; Temp, temperature; Geo, curvilinear distance to the estuary from each site.

N.S., nonsignificant factors removed by the first run of dbRDA. Only the results of the second run are reported.

**Table S9** Envfit results of the dbRDA for relationships between benthic comammox *Nitrospira* and AOP community Bray-Curtis dissimilarity and geographic and environmental variables.

| Variables | Comammox *Nitrospira* | | |  | AOPs | | |
| --- | --- | --- | --- | --- | --- | --- | --- |
|  | CAP1 | CAP2 | *p* |  | CAP1 | CAP2 | *p* |
| NH_3_-N | N.S. | | |  | N.S. | | |
| NO_3_-N | N.S. | | |  | N.S. | | |
| TN | N.S. | | |  | N.S. | | |
| **pH** | N.S. | | |  | -0.939 | -0.344 | 0.0125 |
| **TP** | 0.498 | 0.867 | 0.0440 |  | 0.534 | 0.845 | 0.0100 |
| **TOC** | 0.989 | -0.150 | 0.0053 |  | 0.983 | -0.184 | 0.0022 |
| **Geo** | -0.989 | -0.145 | 0.0001 |  | -0.983 | -0.185 | 0.0001 |

Abbreviations: NH_3_-N, ammonia; NO_3_-N, nitrate; TN, total nitrogen; TP, total phosphorus; TOC, total organic carbon; Geo, curvilinear distance to the estuary from each site.

N.S., nonsignificant factors removed by the first run of dbRDA. Only the results of the second run are reported.

**Table S10** Detailed information for each dominant AOP taxon with a significant difference (*p* < 0.05) in relative abundance between sediments collected immediately upstream and downstream of the TGD. All the relative abundance metrics used are based on the normalized *amoA* gene hits.

| AOPs | Taxonomy^a^ | Relative abundances in the upstream samples | | | | |  | Relative abundances in the downstream samples | | | *p* |
| --- | --- | --- | --- | --- | --- | --- | --- | --- | --- | --- | --- |
|  |  | 13BD_S1 | 13BD_S2 | 14MH_S1 | 14MH_S2 | 14MH_S3 |  | 16YC_S | 18CLJL_S1 | 18CLJL_S2 |  |
| Comammox *Nitrospira* taxa | Clade A-Ib | 12.75 | 10.04 | 26.32 | 11.47 | 37.11 |  | 1.97 | 0 | 0 | 0.0366 |
|  | Clade A-Ib | 4.25 | 8.60 | 11.28 | 11.47 | 15.12 |  | 1.97 | 0 | 0 | 0.0082 |
|  | Clade A-Ic | 52.41 | 25.81 | 42.62 | 21.66 | 52.23 |  | 7.88 | 1.31 | 0 | 0.0066 |
|  | Clade A-Ic | 75.08 | 78.87 | 61.42 | 38.23 | 75.59 |  | 4.93 | 1.31 | 0 | 0.0007 |
|  | Clade A-Ic | 35.41 | 21.51 | 22.56 | 3.82 | 28.86 |  | 1.97 | 0 | 1.37 | 0.0233 |
|  | Clade A-Ic | 18.42 | 17.21 | 8.77 | 6.37 | 20.62 |  | 0 | 0 | 0 | 0.0090 |
|  | Clade A-Ic | 18.42 | 20.08 | 16.30 | 0 | 24.73 |  | 1.97 | 0 | 0 | 0.0353 |
|  | Clade A-Ic | 51.00 | 50.19 | 60.17 | 24.21 | 75.59 |  | 14.78 | 1.31 | 6.83 | 0.0082 |
|  | Clade A-IIb | 17.00 | 7.17 | 13.79 | 12.74 | 26.11 |  | 1.97 | 1.31 | 1.37 | 0.0160 |
|  | Clade B | 11.33 | 5.74 | 8.77 | 10.19 | 10.99 |  | 3.94 | 4.92 | 4.10 | 0.0072 |
| AOB taxa | *Nitrosomonas* (WP_090572320.1) | 1.42 | 4.30 | 3.76 | 3.82 | 6.87 |  | 0 | 0 | 0 | 0.0131 |
|  | *Nitrosomonas* (AEO92104.1) | 12.75 | 12.91 | 6.27 | 2.55 | 5.50 |  | 0 | 0 | 1.37 | 0.0350 |
|  | *Nitrosomonas* (AEJ02636.1) | 35.41 | 21.51 | 31.34 | 15.29 | 19.24 |  | 0.99 | 2.61 | 6.83 | 0.0068 |
|  | *Nitrosomonas* (WP_090544354.1) | 1.42 | 2.87 | 2.51 | 3.82 | 2.75 |  | 0 | 0 | 0 | 0.0131 |
|  | *Nitrosomonas* (AMR34748.1) | 4.25 | 1.43 | 2.51 | 1.27 | 2.75 |  | 0 | 0 | 0 | 0.0142 |
| AOA taxa | *Crenarchaeote* (AFH88881.1) | 0 | 0 | 0 | 0 | 1.37 |  | 0.99 | 3.92 | 4.10 | 0.0162 |
|  | *Crenarchaeote* (ABU40686.1) | 0 | 0 | 1.25 | 0 | 1.37 |  | 1.97 | 3.92 | 6.83 | 0.0159 |
|  | *Nitrososphaera* (AFK24904.1) | 1.42 | 1.43 | 0 | 0 | 0 |  | 1.97 | 2.61 | 4.10 | 0.0121 |
|  | *Nitrososphaera* (BAJ22406.1) | 0 | 0 | 0 | 2.55 | 0 |  | 0.99 | 9.15 | 6.83 | 0.0352 |
|  | *Nitrososphaera* (BAJ22403.1) | 0 | 1.43 | 2.51 | 0 | 0 |  | 1.97 | 9.15 | 5.46 | 0.0290 |
|  | *Nitrososphaera* (AFK24904.1) | 1.42 | 1.43 | 1.25 | 1.27 | 1.37 |  | 3.94 | 3.92 | 5.46 | 0.0002 |
|  | *Nitrosarchaeum* (EGP94363.1) | 7.08 | 12.91 | 11.28 | 25.49 | 17.87 |  | 1.97 | 0 | 1.37 | 0.0171 |
|  | *Nitrosarchaeum* (CCH03865.1) | 0 | 0 | 0 | 0 | 0 |  | 5.91 | 0 | 5.46 | 0.0341 |

^a^Taxonomic affiliations (phylum or genus level) of each AOA and AOB *amoA* gene were directly obtained from NCBI taxonomies or BLAST results against the NCBI reference protein database (best hit: e-value < 10^-5^). The *p* values of one-way ANOVA are provided.

**References**

1. Liu T, Zhang AN, Wang J, Liu S, Jiang X, Dang C, et al. Integrated biogeography of planktonic and sedimentary bacterial communities in the Yangtze River. Microbiome. 2018;6:16.
2. Wang J, Liu Q, Zhao X, Borthwick AGL, Liu Y, Chen Q, et al. Molecular biogeography of planktonic and benthic diatoms in the Yangtze River. Microbiome. 2019;7:153.
3. Zhu G, Wang S, Wang Y, Wang C, Risgaard-Petersen N, Jetten MSM, et al. Anaerobic ammonia oxidation in a fertilized paddy soil. ISME J. 2011;5:1905-12.
4. Daims H, Lebedeva EV, Pjevac P, Han P, Herbold C, Albertsen M, et al. Complete nitrification by *Nitrospira* bacteria. Nature. 2015;528:504-9.
5. Wang Y, Ma L, Mao Y, Jiang X, Xia Y, Yu K, et al. Comammox in drinking water systems. Water Res. 2017;116:332-41.
6. Fu L, Niu B, Zhu Z, Wu S, Li W. CD-HIT: accelerated for clustering the next-generation sequencing data. Bioinformatics. 2012;28:3150-2.
7. Katoh K, Standley DM. MAFFT multiple sequence alignment software version 7: improvements in performance and usability. Mol Biol Evol. 2013;30:772-80.
8. Price MN, Dehal PS, Arkin AP. FastTree 2–approximately maximum-likelihood trees for large alignments. PLoS ONE. 2010;5:e9490.
9. Jones DT, Taylor WR, Thornton JM. The rapid generation of mutation data matrices from protein sequences. Bioinformatics. 1992;8:275-82.
10. Palomo A, Fowler SJ, Gülay A, Rasmussen S, Sicheritz-Ponten T, Smets BF. Metagenomic analysis of rapid gravity sand filter microbial communities suggests novel physiology of *Nitrospira* spp. ISME J. 2016;10:2569-81.
11. Yang Y, Jiang XT, Zhang T. Evaluation of a hybrid approach using UBLAST and BLASTX for metagenomic sequences annotation of specific functional genes. PLoS ONE. 2014;9:e110947.
12. Edgar RC. Search and clustering orders of magnitude faster than BLAST. Bioinformatics. 2010;26:2460-1.
13. Ma L, Xia Y, Li B, Yang Y, Li LG, Tiedje JM, et al. Metagenomic assembly reveals hosts of antibiotic resistance genes and the shared resistome in pig, chicken, and human feces. Environ Sci Technol. 2015;50:420-7.
14. Zhu W, Lomsadze A, Borodovsky M. Ab initio gene identification in metagenomic sequences. Nucleic Acids Res. 2010;38:e132.
15. Buchfink B, Xie C, Huson DH. Fast and sensitive protein alignment using DIAMOND. Nat Methods. 2015;12:59-60.
16. Huson DH, Auch AF, Qi J, Schuster SC. MEGAN analysis of metagenomic data. Genome Res. 2007;17:377-86.
17. Albertsen M, Hugenholtz P, Skarshewski A, Nielsen KL, Tyson GW, Nielsen PH. Genome sequences of rare, uncultured bacteria obtained by differential coverage binning of multiple metagenomes. Nat Biotechnol. 2013;31:533-8.
18. Parks DH, Imelfort M, Skennerton CT, Hugenholtz P, Tyson GW. CheckM: assessing the quality of microbial genomes recovered from isolates, single cells, and metagenomes. Genome Res. 2015;25:1043-55.
19. Tatusov RL, Galperin MY, Natale DA, Koonin EV. The COG database: a tool for genome-scale analysis of protein functions and evolution. Nucleic Acids Res. 2000;28:33-6.
20. Kanehisa M, Goto S. KEGG: kyoto encyclopedia of genes and genomes. Nucleic Acids Res. 2000;28:27-30.
21. Capella-Gutiérrez S, Silla-Martínez JM, Gabaldón T. trimAl: a tool for automated alignment trimming in large-scale phylogenetic analyses. Bioinformatics. 2009;25:1972-3.
22. Darling AE, Jospin G, Lowe E, Matsen IV FA, Bik HM, Eisen JA. PhyloSift: phylogenetic analysis of genomes and metagenomes. PeerJ. 2014;2:e243.
23. Kumar S, Stecher G, Tamura K. MEGA7: molecular evolutionary genetics analysis version 7.0 for bigger datasets. Mol Biol Evol. 2016;33:1870-4.
24. Hua ZS, Qu YN, Zhu Q, Zhou EM, Qi YL, Yin YR, et al. Genomic inference of the metabolism and evolution of the archaeal phylum Aigarchaeota. Nat Commun. 2018;9:2832.
25. Whelan S, Goldman N. A general empirical model of protein evolution derived from multiple protein families using a maximum-likelihood approach. Mol Biol Evol. 2001;18: 691-9.
26. Lyu Z, Lu Y. Metabolic shift at the class level sheds light on adaptation of methanogens to oxidative environments. ISME J. 2018;12:411-23.
27. Kits KD, Sedlacek CJ, Lebedeva EV, Han P, Bulaev A, Pjevac P, et al. Kinetic analysis of a complete nitriﬁer reveals an oligotrophic lifestyle. Nature. 2017;549:269-72.
28. Richter M, Rosselló-Móra R. Shifting the genomic gold standard for the prokaryotic species definition. Proc Natl Acad Sci USA. 2009;106:19126-31.
29. van Kessel MAHJ, Speth DR, Albertsen M, Nielsen PR, Op den Camp HJM, Kartal B, et al. Complete nitrification by a single microorganism. Nature. 2015;528:555-9.
30. Camejo PY, Santo Domingo J, McMahon KD, Noguera DR. Genome-enabled insights into the ecophysiology of the comammox bacterium “*Candidatus* Nitrospira nitrosa”. mSystems. 2017;2:e00059-17.
31. Palomo A, Pedersen AG, Fowler SJ, Dechesne A, Sicheritz-Pontén T, Smets BF. Comparative genomics sheds light on niche differentiation and the evolutionary history of comammox *Nitrospira*. ISME J. 2018;12:1779-93.
32. Pinto AJ, Marcus DN, Ijaz UZ, Bautista-de lose Santos QM, Dick GJ, Raskin L. Metagenomic evidence for the presence of comammox *Nitrospira*-like bacteria in a drinking water system. mSphere. 2016;1:e00054-15.
33. Ehrich S, Behrens D, Lebedeva E, Ludwig W, Bock E. A new obligately chemolithoautotrophic, nitrite-oxidizing bacterium, *Nitrospira* moscoviensis sp. nov. and its phylogenetic relationship. Arch Microbiol. 1995;164:16-23.
34. Ushiki N, Fujitani H, Aoi Y, Tsuneda S. Isolation of *Nitrospira* belonging to sublineage II from a wastewater treatment plant. Microbes Environ. 2013;28:346-53.
35. Speth DR, in ’t Zandt MH, Guerrero-Cruz S, Dutilh BE, Jetten MSM. Genome-based microbial ecology of anammox granules in a full-scale wastewater treatment system. Nat Commun. 2016;7:11172.
36. Spieck E, Hartwig C, McCormack I, Maixner F, Wagner M, Lipski A, et al. Selective enrichment and molecular characterization of a previously uncultured *Nitrospira*-like bacterium from activated sludge. Environ Microbiol. 2006;8:405-15.
37. Fujitani H, Ushiki N, Tsuneda S, Aoi Y. Isolation of sublineage I *Nitrospira* by a novel cultivation strategy. Environ Microbiol. 2014;16:3030-40.
38. Slaby BM, Hackl T, Horn H, Bayer K, Hentschel U. Metagenomic binning of a marine sponge microbiome reveals unity in defense but metabolic specialization. ISME J. 2017;11:2465-78.
